# Interaction between the microbiome and TP53 in human lung cancer

**DOI:** 10.1186/s13059-018-1501-6

**Published:** 2018-08-24

**Authors:** K. Leigh Greathouse, James R. White, Ashely J. Vargas, Valery V. Bliskovsky, Jessica A. Beck, Natalia von Muhlinen, Eric C. Polley, Elise D. Bowman, Mohammed A. Khan, Ana I. Robles, Tomer Cooks, Bríd M. Ryan, Noah Padgett, Amiran H. Dzutsev, Giorgio Trinchieri, Marbin A. Pineda, Sven Bilke, Paul S. Meltzer, Alexis N. Hokenstad, Tricia M. Stickrod, Marina R. Walther-Antonio, Joshua P. Earl, Joshua C. Mell, Jaroslaw E. Krol, Sergey V. Balashov, Archana S. Bhat, Garth D. Ehrlich, Alex Valm, Clayton Deming, Sean Conlan, Julia Oh, Julie A. Segre, Curtis C. Harris

**Affiliations:** 1 Laboratory of Human Carcinogenesis, Center for Cancer, Research, National Cancer Institute, National Institutes of Health, 37 Convent Dr., Rm 3068A, MSC 4258, Bethesda, MD 20892-4258 USA; 2 Resphera Biosciences, Baltimore, MD 21231 USA; 3 Center for Cancer Research Genomics Core, National Cancer Institute, National Institutes of Health, Bethesda, MD 20892 USA; 4 Division of Biomedical Statistics and Informatics, Mayo Clinic, Rochester, MN 55905 USA; 5 Department of Educational Psychology, Baylor University, Waco, TX 97346 USA; 6 Laboratory of Experimental Immunology, Center for Cancer Research, National Cancer Institute, National Institutes of Health, Bethesda, MD 20892 USA; 7 Genetics Branch, Center for Cancer Research, National Cancer Institute, National Institutes of Health Bethesda, Bethesda, MD 20892 USA; 8 Department of Obstetrics and Gynecology, Mayo Clinic, Rochester, MN USA; 9 Microbiome Laboratory, Mayo Clinic, Rochester, MN 55905 USA; 10 Department of Surgery, Mayo Clinic, Rochester, MN 55905 USA; 11 Department of Microbiology and Immunology, Center for Genomic Sciences, Institute of Molecular Medicine and Infectious Disease, Drexel University College of Medicine, Philadelphia, PA 19129 USA; 12 National Human Genome Research Institute, National Institutes of Health, Bethesda, MD 20892 USA; 13 Jackson Laboratory, Framingham, CT 06032 USA; 14 Present Address: Nutrition Sciences, Baylor University, Waco, TX 97346 USA

**Keywords:** Lung cancer, Microbiome, TP53, Squamous cell carcinoma, Mutation

## Abstract

**Background:**

Lung cancer is the leading cancer diagnosis worldwide and the number one cause of cancer deaths. Exposure to cigarette smoke, the primary risk factor in lung cancer, reduces epithelial barrier integrity and increases susceptibility to infections. Herein, we hypothesize that somatic mutations together with cigarette smoke generate a dysbiotic microbiota that is associated with lung carcinogenesis. Using lung tissue from 33 controls and 143 cancer cases, we conduct 16S ribosomal RNA (rRNA) bacterial gene sequencing, with RNA-sequencing data from lung cancer cases in The Cancer Genome Atlas serving as the validation cohort.

**Results:**

Overall, we demonstrate a lower alpha diversity in normal lung as compared to non-tumor adjacent or tumor tissue. In squamous cell carcinoma specifically, a separate group of taxa are identified, in which *Acidovorax* is enriched in smokers. *Acidovorax* temporans is identified within tumor sections by fluorescent in situ hybridization and confirmed by two separate 16S rRNA strategies. Further, these taxa, including *Acidovorax*, exhibit higher abundance among the subset of squamous cell carcinoma cases with TP53 mutations, an association not seen in adenocarcinomas.

**Conclusions:**

The results of this comprehensive study show both microbiome-gene and microbiome-exposure interactions in squamous cell carcinoma lung cancer tissue. Specifically, tumors harboring TP53 mutations, which can impair epithelial function, have a unique bacterial consortium that is higher in relative abundance in smoking-associated tumors of this type. Given the significant need for clinical diagnostic tools in lung cancer, this study may provide novel biomarkers for early detection.

**Electronic supplementary material:**

The online version of this article (10.1186/s13059-018-1501-6) contains supplementary material, which is available to authorized users.

## Background

Lung cancer is the leading cancer diagnosis worldwide (1.8 million/year) and has a higher mortality than that of the next top three cancers combined (158,080 vs 115,760 deaths) [1]. Unfortunately, lung cancer survival remains poor and has shown minimal improvement over the past five decades, owing to diagnosis at advanced stage and resistance to standard chemotherapy [2]. While we have made significant strides with targeted receptor therapy and immunotherapy, biomarkers with higher specificity would improve diagnosis and treatment for these individuals.

Epidemiological evidence indicates an association between repeated antibiotic exposure and increased lung cancer risk; however, the contribution of the lung microbiome to lung cancer is unknown [3]. The first line of defense against inhaled environmental insults, including tobacco smoke and infection, is the respiratory epithelium. Until recently, healthy lungs were regarded as essentially sterile; however, studies now illustrate the presence of a lung microbiota [4], the community of microscopic organisms living within the host lung, which is altered in respiratory diseases including asthma, chronic obstructive pulmonary disease (COPD), and cystic fibrosis [5]. Disruption of the epithelium by tobacco smoke can be a primary cause of inflammatory pathology, which is seen in both COPD and lung cancer. Dysbiosis has been observed in both humans and model systems of COPD and cystic fibrosis [6, 7]. In COPD patients and in vitro, cigarette smoke has been shown to reduce epithelial integrity and cell–cell contact, which can increase susceptibility to respiratory pathogens or other environmental pollutants [8]. Disturbances in the microbiome, from cigarette smoke, epithelial damage, or gene mutations, can allow pathogenic species to dominate the community or increase virulence of other normally commensal microbes. Evidence of this has been demonstrated in patients with cystic fibrosis who have more virulent forms of *P. aeruginosa* [9]. These inflammatory associated events have been proposed to lead to an increased risk or progression of diseases, including lung cancer.

Several bacteria are associated with chronic inflammation and subsequent increased risk of lung and colon cancer, including *Mycobacterium tuberculosis* (lung cancer) [10], *Bacteroides fragilis*, and *Fusobacterium nucleatum* (colon cancer) [11]. Recent microbiome studies in colon cancer have demonstrated a contribution of bacteria to carcinogenesis. Specifically, *F. nucleatum*, a bacterium commonly isolated from patients with inflammatory bowel disease, may be a risk factor for colon cancer [11, 12]. The more virulent strains of *F. nucleatum* affect colon cancer progression in animal models and increase tumor multiplicity [13] by various mechanisms including favoring the infiltration of tumor-promoting myeloid cells to create a pro-inflammatory environment [14]. Colorectal carcinomas associated with high abundance of fecal *F. nucleatum* were found to have the highest number of somatic mutations, suggesting that these mutations create a pathogen-friendly environment [15]. Similarly, *B. fragilis* can secrete endotoxins that cause DNA damage leading to mutations and colon cancer initiation [16]. Furthermore, the loss of the oncogenic protein p53 in enterocytes impairs the epithelial barrier and allows infiltration of bacteria resulting in inflammatory signaling (NF-κB), which is required for tumor progression [17]. The tumor suppressor gene *TP53* is the most commonly mutated gene in lung cancer [18], with certain missense mutations showing gain of oncogenic function [19]; however, the relationship between *TP53* and microbiota in lung cancer remains unknown. Herein, we hypothesize that somatic mutations together with environmental exposures are correlated with tissue-associated alterations in the microbial community of the lung, which may participate in lung carcinogenesis.

## Results

To investigate the lung mucosal-associated microbial alterations in the etiology of lung cancer, we analyzed samples from the NCI-MD case-control study (*n* = 143 tumor and *n* = 144 non-tumor adjacent tissues) and lung cancer samples from The Cancer Genome Atlas (TCGA; *n* = 1112 tumor and non-tumor adjacent RNA-sequencing [RNA-seq] data from tissues) for validation. In addition, we used the clinical information from these two sample populations to control for confounders in lung cancer risk and progression (age, gender, smoking, race, family and medical history, and co-morbidities), as well as factors that are known to alter the human microbiome (antibiotics and neoadjuvant therapy). Given the paucity of healthy lung tissue available for study, we utilized two separate tissue biorepositories. Non-cancerous lung tissue was obtained by lung biopsy from individuals with benign lung nodules without cancer or non-cancer lung from immediate autopsy [20], which was used as a referent control (Table 1).

**Table 1:** Descriptive summary of population samples

	Control lung		NCI-MD study		TCGA study	
	ImA	HB^a^	Normal adjacent	Tumor	Normal adjacent	Tumor
	(*n* = 33)	(*n* = 16)	(*n* = 144)	(*n* = 143)	(*n* = 108)	(*n* = 974)
Age - mean (SD)	39.5 (18.8)	62.6 (7.7)	65.5 (9.8)	65.7 (9.9)	66.9 (9.9)	66.4 (9.2)
< Mean	18	9	70	63	49	396
≥ Mean	15	7	74	80	59	578
Unknown					5	128
Gender						
M	25	11	92	87	58	514
F	8	5	52	56	45	355
Unknown					5	105
Race^b^						
EA	27	14	86	95	90	650
AA	5	2	58	48	8	42
Other						59
Unknown	1				10	223
Smoking status^b^						
Ever		14	122	127	90	768
Former		11	44	40	71	551
Current		3	64	70	19	217
Never		2	9	7	7	120
Unknown						
Stage						
I (a/b)				69	52	454
II (a/b)				44	28	231
III (a/b)				11	19	155
IV				2	3	29
Unknown				16	6	105
Histology						
AD				67	58	485
SCC				47	50	489
Other				29		
TP53 mutation status						
Wild-type (AD/SCC)				32/11		125/59
Mutant (AD/SCC)				29/35		104/118
Unknown				36		568

^a^Two cases removed due to emphysema

^b^Smoking status and race self-reported

*ImA* immediate autopsy, *HB* hospital biopsy

Given the high potential for contamination in low-biomass samples, such as the lung, we took several measures to address this issue controlling for contamination points in the collection process. To assess possible confounding with sequence quality, we conducted sequencing quality control analysis by Phred score and by sequencing run (Additional file 1: Figure S1). In order to remove possible contaminants from our analysis, we first performed a threshold analysis similar to a previous study [21], wherein we plotted the mean percent abundance across experimental samples versus negative control samples and removed those that were ≥ 5% in both experimental and negative control samples (Additional file 1: Figure S2). We next applied a statistical analysis wherein we used a systematic removal process of putative contaminants including *Herbaspirillum*, *Halomonas*, and *Shewanella* (Additional file 1: Table S1). At each stage of removal, we report the number of Mann–Whitney *p* values < 0.05 comparing paired tumor normal samples showing the greatest rise the number of significant p-values with the removal top five contaminants (Additional file 1: Table S1). At each stage of removal, we report the number of Mann–Whitney *p* values < 0.05 comparing paired tumor normal samples showing the greatest rise the number of significant *p* values with the removal top five contaminants (Additional file 1: Table S1). Additionally, we conducted hierarchal clustering of negative controls, non-tumor samples, and tumor samples independently in order to visualize and identify the strongest sources of contamination (Additional file 1: Figures S2 and S3). The combination of these analyses resulted in initial removal of the genera *Halomonas*, *Herbaspririllium*, *Shewanella*, *Propionibacterium*, and *Variovorax*.

To identify the microbial communities present in each tissue type, we sequenced the V3–V5 16S ribosomal RNA (rRNA) bacterial gene using the Illumina MiSeq platform. After quality filtering and contaminant removal, 34 million quality sequences were retained for operational taxonomic unit (OTU) clustering and downstream analysis (Additional file 1: Table S2).

To enable us to validate findings from our NCI-MD 16S rRNA gene sequencing analysis, we took advantage of the TCGA lung cancer database. Using the unmapped RNA-seq reads from these samples (*N* = 1112 and *n* = 106 paired tumor/non-tumor), we analyzed with our metagenomics analysis pipeline. After removal of all human reads, we took the remaining non-human reads and used three separate tools, MetaPhlAn, Kraken, and PathoScope, to assign reads to taxonomy, including bacteria, virus, and fungi (Additional file 1: Table S2). Due to the highly curated database of PathoScope, we were able obtain to species and in some cases strain-level putative identification of RNA-seq reads. For this reason, and due to its rigorous validation in other studies [22], we used these data as our validation dataset. Unfortunately, given that all patients in this database had lung cancer, we could not validate our microbial findings in non-diseased lung tissue in the TCGA dataset. Given that this was one of the first times TCGA was used to completely profile the microbiota of lung cancer, we asked how similar the 16S rRNA gene sequencing and RNA-seq microbial communities were at the phylum and genus levels. Using an overall threshold of 0.01% of genus level abundance, we identified 236 overlapping genera out of 520 total genera in the 16S rRNA gene sequencing data and 609 total genera in the RNA-seq data (Additional file 1: Figure S4).

### Bacterial profile of the lung cancer microbiome is dominated by Proteobacteria and validated in a separate lung cancer data set

**Fig. 1:**
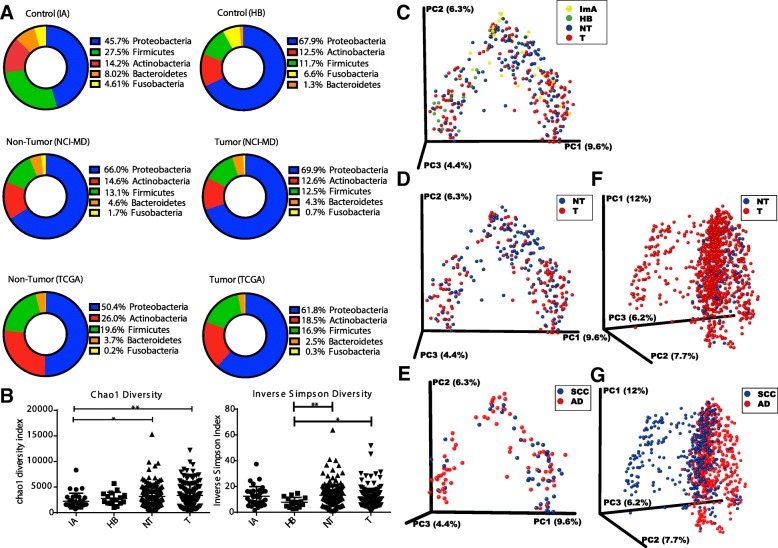
The bacterial profile and diversity of the lung microbiome in non-diseased and cancerous tissues. **a** 16S rRNA gene sequences from non-diseased lung (ImA or HB; *top*), non-tumor adjacent (NT) and tumor (T) assigned to OTUs or proportional abundance of metatranscriptomic sequences (TCGA; *bottom*) at the phylum level showing the most dominant taxa for each tissue type. **b** Alpha diversity between non-diseased lung tissue (ImA and HB) non-tumor adjacent (NT) and tumors from 16S rRNA gene sequencing using Chao1 (richness) or inverse Simpson index. *p < 0.05, **p < 0.01. Test of significance is Mann–Whitney. PCoA plots from NCI-MD study of tissue microbiome beta-diversity colored by (**c**) all tissue types, (**d**) cancer cases, and (**e**) histological subtype; and from TCGA study of (**f**) cancer cases and (**g**) histological subtype. ImA immediate autopsy, HB hospital biopsy

We know from previous microbial studies of lung disease that bacterial composition shifts occur compared to normal non-diseased lungs [23] and associated with disease severity [24]; however, these compositional changes have not been examined in lung cancer. In order to identify the microbial changes associated with lung cancer, we first examined the ecological diversity within samples (alpha diversity) and between samples (beta diversity) of non-cancerous (immediate autopsy and hospital biopsy) tissues, non-tumor adjacent (NT) and tumor (T) tissues from 16S rRNA gene sequencing. At the phylum level, we observed increases in Proteobacteria (Kruskal–Wallis *p =* 0.0002) and decreases in Firmicutes (Kruskal–Wallis *p =* 0.04) in lung tissue hospital biopsies, as well as in tumor and associated non-tumor tissues from the NCI-MD study compared with non-cancer population control lung tissues, as has been seen in COPD [25] (Fig. 1a). Further, we note higher Fusobacterium in ImA and HB controls as compared to cancer cases, though it is unclear what this finding indicates at the phylogenic level. We also observed a similar increase in Proteobacteria (Mann–Whitney *p =* 0.02) between non-tumor lung tissue and lung cancer in the TCGA study, indicating that this is recurrent phenomenon in lung cancer (Fig. 1a). However, the lack of similarity between the NCI-MD and TCGA non-tumor samples may be attributed to the TCGA data being derived from multiple sample populations in the United States, differences in sample prep and in sequencing platforms, as illustrated by Meisel et al. [26].

To identify ecological diversity changes associated with lung cancer, we next examined the richness (Chao1) and diversity (Inverse Simpson) of the microbiome within samples (alpha diversity) of non-disease (immediate autopsy and hospital biopsy) lung tissues, non-tumor adjacent tissues, and tumor tissues from 16S rRNA gene sequencing (NCI-MD study). Specifically, Chao1 measurement demonstrated a significant increase in both tumor and non-tumor tissue richness as compared to immediate autopsy control tissue samples (Fig. 1b). Similarly, using the Inverse Simpson index, which measures number (richness) and abundance (evenness) of species, we observed a significant increase in alpha diversity in both tumor and non-tumors as compared to hospital biopsy control tissues (Fig. 1b), similar to studies of severe COPD [27], indicating that microbial diversity of lung cancer tissues is altered from its non-diseased state. When we examined tissue from cancer cases, alpha diversity was significantly different between tumor and non-tumors in the NCI-MD study and TCGA study, but results were not consistent between studies or diversity metrics (Additional file 1: Figure S5). However, we did not see any significant changes in alpha diversity by smoking status (never, former, or current) nor correlation with time since quitting smoking (Additional file 1: Figure S4), in cancer-free or lung cancer tissues as has been demonstrated in other lung microbiome studies [28, 29].

We also asked whether there were differences between microbial communities using beta diversity (Bray Curtis). Since we were comparing between studies and between types of sequencing (16S rRNA and RNA-seq), we used a method that could be commonly applied between studies, which excludes phylogeny (e.g. Bray Curtis). Within the NCI-MD study, we observed significant differences in beta diversity between all tissue types (PERMANOVA *F* = 2.90, *p =* 0.001), tumor and non-tumor (PERMANOVA *F* = 2.94, *p =* 0.001), and adenocarcinoma (AD) versus squamous cell carcinoma (SCC) (PERMANOVA *F* = 2.27, *p =* 0.005), with tumor vs. non-tumor having the largest among-group distance denoted by the higher *F* value (Fig. 1c–e). Similarly, we observed significant difference in beta diversity between tumor and non-tumor (PERMANOVA *F* = 3.63, *p =* 0.001) and AD v SCC (PERMANOVA *F* = 27.19, *p =* 0.001) (Fig. 1f, g). Together, these data illustrate a trend of increasing diversity and richness associated with lung cancer.

### A distinct group of taxa are enriched in squamous cell carcinoma with *Acidovorax* more abundant in smokers

**Fig. 2:**
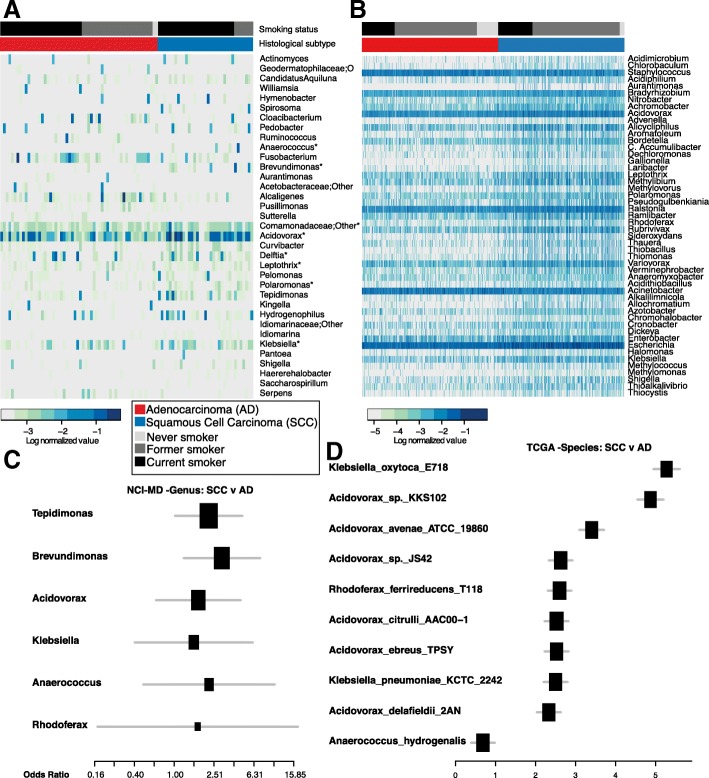
Taxonomic consortia differentiating smoking status and histological subtype of lung cancer. **a** *Heat maps* showing top differentially abundant genera (NCI-MD) (Mann–Whitney *p* value < 0.05; * overlapping between NCI-MD and TCGA) between AD and SCC lung cancer tissue sorted by histological subtype and smoking status. **b** *Heat map* showing genera (TCGA) that that are differentially abundant between AD and SCC (Mann–Whitney FDR corrected *p* < 0.05), sorted by histological subtype and smoking. **c** *Forest plot* of odds ratios for genera in NCI-MD dataset that are significantly associated with SCC compared to AD in tumors (adjusted odds ratio *p* < 0.05). **d** *Forest plot* of odds ratios for species in the TCGA dataset that are significantly associated with SCC vs AD in tumors (adjusted odds ratio FDR corrected *p* < 0.05)

The two most common types of non-small cell lung cancer are SCC and AD, arising centrally from the cells lining the bronchi and from peripheral airways, respectively. Previous studies report that the microbial community differs between the bronchi and lower lungs in COPD [6]. This phenomenon of anatomic-specific microbial variation was also apparent in the abundance of genera between bronchial and SCC tumors from the upper lungs with higher abundance of *Acidovorax* in comparison to AD tumors (Additional file 1: Figure S6). Further, the taxonomic distribution in AD tumors appears more similar to the taxonomic abundance in COPD, which is generally dominated by *Pseudomonas* [6]. Given this distinction, we controlled for this potential confounder of lung location in subsequent analyses. This led us to investigate the specific taxonomic pattern further and ask if there was a specific microbial consortia that is enriched in SCC or AD tumor tissue. In the NCI-MD study, we identified 32 genera that were differentially abundant in SCC (*n* = 47) versus AD (*n* = 67) tumors (Student’s t-test; MW *P* < 0.05), nine of which were significant after multiple testing correction (FDR) (*Acidovorax*, *Brevundimonas*, *Comamonas*, *Tepidimonas*, *Rhodoferax*, *Klebsiella*, *Leptothrix*, *Polaromonas*, *Anaerococcus*) (Fig. 2a). We also validated these same observations in the TCGA dataset (AD = 485, SCC = 489) (Mann–Whitney FDR corrected *p* value< 0.05) (Fig. 2b). To control for potential confounders of this association, including age, gender, race, smoking, anatomical location, and stage, we conducted adjusted logistic regression analysis in the NCI-MD study for each taxa separately and confirmed 6/9 of these genera were significantly associated with increased odds of being SCC as compared to AD lung cancer (Fig. 2c, Additional file 1: Tables S5 and S7). Though we had reduced power, we asked whether the time since quitting smoking would change this association, and found that *Acidovorax*, *Klebsiella*, *Tepidimonas*, *Rhodoferax*, and *Anaerococcus* remained significant. When we examined the larger TCGA dataset, we also found significantly increased odds of being SCC as compared with AD among 4/9 (*Acidovorax*, *Klebsiella*, *Rhodoferax*, *Anaerococcus*) of the same genera in adjusted models (FDR corrected *P* < 0.05) (Fig. 2d, Additional file 1: Tables S6 and S8). This association also remained significant after adjusting for pack years and time since quitting smoking. Together these data, validated in two separate cohorts, demonstrate that a specific community of taxa is more abundant in SCC as compared with AD lung cancer tissue, and are capable of distinguishing between AD and SCC tumors from individuals with similar exposure to cigarette smoke. However, whether this is a cause or consequence of the development of SCC cancer remains unknown.

**Fig. 3:**
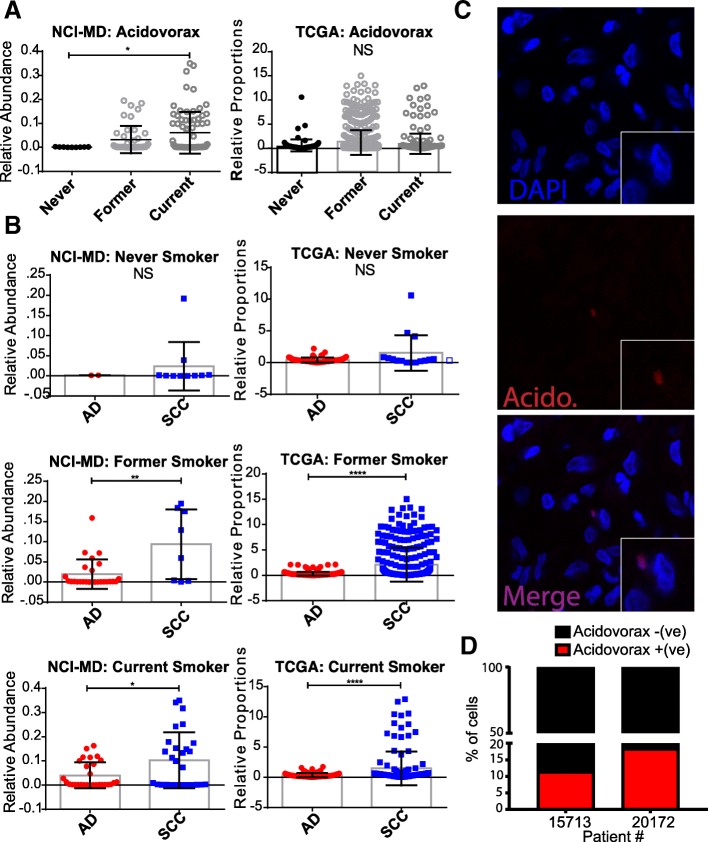
Relative abundance of *Acidovorax* stratified by smoking status and histological subtype. **a** Relative abundance of *Acidovorax* stratified by smoking status in the NCI-MD (*left*) and TCGA (*right*) datasets. **b** Relative abundance of *Acidovorax* in never, former, and current smokers stratified by histological subtype in the NCI-MD (*left*) and TCGA (*right*) datasets. **c** Representative FISH images of tumor tissue sections using fluorescent probe specific to *Acidovorax*. **d** Quantification of *Acidovorax* probe reactivity (10 fields; at least 300 cells counted) showing percentage (%) of cells with perinuclear probe reactivity from two lung cancer cases (15,713 – SCC/current smoker; 20,172 – SCC/former smoker). *p < 0.05, **p < 0.01, ****p < 0.0001. Tests of significance are Mann–Whitney or Kruskal–Wallis and Dunn’s multiple comparisons test. NS non-significant


Both SCC and AD lung cancers are associated with smoking; however, the association between smoking and SCC is stronger [30], which leads us to ask whether any of the SCC-enriched taxa were also associated with smoking. We stratified the tumor samples into never smokers (*n* = 7) or ever-smokers (current [*n* = 70] and former smokers [*n* = 40]) using linear discriminant analysis (LEfSe) to identify smoking-associated microbial biomarkers in SCC tumors. We identified six genera that were able to distinguish ever (former and current) versus non-smokers in our NCI-MD study (*Acidovorax*, *Ruminococcus*, *Oscillospira*, *Duganella*, *Ensifer*, *Rhizobium*) (Additional file 1: Figure S6C). Specifically, *Acidovorax* was more abundant in former and current smokers as compared with never smokers (Kruskal–Wallis *p* value < 0.05) (Fig. 3a), with a similar trend observed in the TCGA dataset (n_never_ = 120, n_former_ = 551, n_current_ = 217) (Kruskal–Wallis *p* = 0.27; ANOVA *p* = 0.02). We did not, however, observe any correlation between *Acidovorax* abundance and smoking time cessation. Interestingly, the relative abundance of *Acidovorax* and *Klebsiella* were higher in former and current smokers when we stratified by histological subtype in both the NCI-MD and TCGA datasets (Fig. 3b, Additional file 1: Figure S7), indicating not only are there bacteria which have a higher relative abundance in tumors from individuals who smoke, but SCC tumors from smokers have even greater relative abundance of these bacteria. We also demonstrated the presence of this bacterium in lung tumors using FISH (Fig. 3c, d, Additional file 1: Figure S8, Additional file 2), and using PacBio sequencing, which identified the species as *A. temperans* (Additional file 1: Table S4). We did not find any significant associations between pack years or time since quitting smoking and the abundance of these taxa in either study among SCC tumors in either study.

**Additional file 2:** Video S1. 3D video image of *Acidovorax*. (MP4 6568 kb)


### *TP53* mutations are associated with enrichment of SSC-enriched taxa

**Fig. 4:**
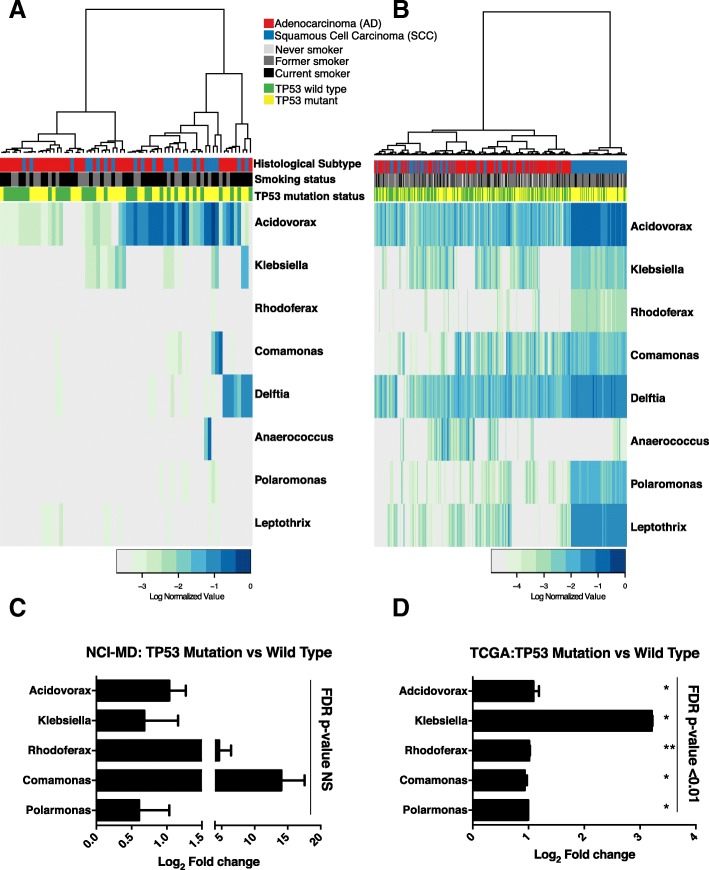
Mutations in *TP53* associated with abundance of taxonomic signature specific to squamous cell lung tumors. **a** *Heat map* of genus-level abundance in NCI-MD data colored by mutation status, *TP53* wild-type or mutated, smoking, and histological subtype in all lung tumor samples. **b** *Heat map* of genus-level abundance from TCGA data in all tumors colored by mutation status, *TP53* wild-type or mutated, smoking, and histological subtype. **c**, **d** Fold change in mean abundance of SCC-associated taxa in NCI-MD or TCGA tissues comparing *TP53* mutated to wild-type. Test of significance is Mann–Whitney. Fold change among all taxa in (**d**) are significant after FDR correction < 0.01. (NCI-MD; SCC_wt_ = 11, SCC_mut_ = 35 and TCGA; SCC_wt_ = 59, SCC_mut_ = 118)

The most prevalent somatic mutation in SCC lung tumors is in the gene *TP53* [31]. Previous studies demonstrate that mutations in *TP53*, specifically in colon cancer, lead to disruption of the epithelial barrier allowing the infiltration of tumor-foraging bacteria and resulting in disease progression [17]. Given that *TP53* mutations are found in 75–80% of SCC tumors, we hypothesized that these SCC-associated taxa may be more abundant in tumors with *TP53* mutations, owing to the loss of the epithelial barrier function in these tumors. To address this question, we investigated the association between *TP53* mutations in both the NCI-MD (*n* = 107) and TCGA (*n* = 409) datasets using either *TP53* specific sequencing (MiSeq) or the published *TP53* mutation analysis data from TCGA [31]. We first analyzed all tumors in the NCI-MD study regardless of histology and identified a group of taxa that were more abundant in tumors with *TP53* mutations (Fig. 4a). To have greater power, we performed the same analysis in the TCGA dataset and observed a significant increase in these same taxa (MW FDR corrected *P* < 0.05) (Fig. 4b). When analyzing only SCC tumors (*n* = 46), this signature became stronger in tumors with *TP53* mutations in both datasets, specifically among the SCC-associated taxa previously identified (Fig. 4c, d). In the NCI-MD study, we found that 5/9 of the genera (*Acidovorax*, *Klebsiella*, *Rhodoferax*, *Comamonas*, and *Polarmonas*) that differentiated SCC from AD were also more abundant in the tumors harboring *TP53* mutations, though not statistically significant (Fig. 4c). In the TCGA dataset, the fold change in all five SCC-associated genera were significantly higher in SCC tumors (*n* = 177) with *TP53* mutations (MW corrected FDR < 0.01; Fig. 4d). Furthermore, using these same SCC-associated taxa we observed no pattern of association in AD tumors with *TP53* mutations indicating this signature was specific to SCC with *TP53* mutations (Additional file 1: Figures S9A and S9B). Overall, these data are consistent with the hypothesis that mutations in *TP53* are associated with the enrichment of a microbial consortia that are highly represented in SCC tumors.

## Discussion

Gene-environment interactions have been identified as contributors to cancer incidence [32]; however, little is known about gene-microbiome interactions in carcinogenesis. We demonstrate a gene-microbiome association in human lung cancer as well as histological evidence of a smoking-associated bacterium, *Acidovorax*. Herein, we identify a microbial consortia that is associated with a histological subtype of lung cancer, SCC, which is further enriched in tumors with mutations in *TP53*. Given the strong association between smoking and development of SCC, it follows that a subgroup of this SCC consortium would also be found in smoking-associated SCC. We validate this assumption finding *Acidovorax spp.* more abundant in SCC tumors harboring *TP53* mutations and confirmed the presence of this genus histologically. These results suggest that smoking together with tumorigenesis may provide an environment conducive to the growth of *Acidovorax spp*. and similar species, which can flourish in nutrient-depleted environments, such as that of the lung. Collectively, these observations indicate that a state of dysbiosis exists in lung cancer. The hypothesis generated is that epithelial cells in the lung exposed to tobacco smoke and/or mutations in *TP53* are invaded by species that take advantage of this new microenvironment, suggesting these bacteria could act as promoters in lung tumorigenesis.

Several cancers are caused by bacteria and viruses, including cervical cancer (HPV), liver cancer (HBV), and gastric cancer (*H. pylori* and potentially *B. fragilis*); however, very few microbes have been identified as carcinogenic. Beyond acting as initiators, other relationships exist between microbe and host tissue environments, in a similar manner to chemical carcinogens. These relationships include bacteria that act as promoters and those that are just passengers in the tumorigenesis process. While this study is not longitudinal, our data suggest the latter two possibilities, either they are promoters or passengers.

In support of the promoter hypothesis, it is plausible that smoking creates an environment that allows these bacteria to outcompete other species for resources and thus survival, which allows exposure to microbial factors enhancing tumorigenesis. Smoking is most strongly associated with the SCC histological subtype of lung cancer; however, whether smoking alters the lung *tissue* microbiome is still not well understood, especially in the context of disease. Multiple studies using various samples tissue and non-tissue types (e.g. oral and/or nasal swabs, bronchial lavage fluid, or lung tissue) have found inconsistent results in alpha diversity by smoking status. From our study, while we did not observe differences in alpha diversity, we observe a significant difference in the taxonomic consortia among smokers as compared to non-smokers, specifically in *Acidovorax* and *Klebsiella spp*. Similarly, oral and nasal microbial taxa differences have been observed between smokers and non-smokers [29, 33]. From a large study of the naso- and oropharynx, significant differences in specific microbial taxa were identified between smokers and non-smokers [34]. Additionally, in a study of non-malignant lung tissue (*n* = 152), they observed a significant increase in alpha diversity with higher number of pack years of smoking [35]. While they identified *Acidovorax*, *Anaerococcus*, and *Comamonas* in smokers, these taxa did not differentiate smokers and non-smokers in a *healthy* population. However, in a recent study of non-malignant lung tissue, which compared tissue to isolated extracellular vesicles (EVs) from tissues, the greater diversity was identified specifically in EVs, with a greater abundance of *Acidovorax* specifically found in the EVs of smokers, indicating a possible factor in differential findings observed among previous studies [36].

These data indicate that smoking alone may be insufficient to alter the microbial population in a healthy population. However, smoking has been shown to suppress the immune system and induce epithelial barrier dysfunction [37]. Specifically, *Acidovorax spp.* have been identified in two common brands of cigarettes [38] and have the capacity to metabolize multiple organic pollutants like those found in cigarette smoke [39]. Therefore, degradation of tobacco smoke compounds, such as polycyclic aromatic hydrocarbons by *Acidovorax spp*., may promote survival of transformed cells and subsequently tumor promotion. These factors may allow taxa direct access to epithelial cells where microbial toxins or reactive oxygen/nitrogen from the aforementioned species to directly or indirectly encourage malignant transformation of the lung epithelium via DNA damage and mutations in *TP53* [40–42]. Once the epithelial barrier defense is lost as a consequence of mutations in *TP53* and malignant transformation, these species then may become tumor-foraging bacteria. In support, several bacterial species have been shown to modulate the tumor-suppressor p53 at both the protein and DNA level [43]. Specifically, the loss of p53 in enterocytes in murine models impairs the epithelial barrier and allows infiltration of bacteria resulting in NF-κB signaling, which was required for tumor progression [17]. This evidence suggests that SCC tumors with *TP53* mutations could have poor epithelial barrier function, thus allowing tumor foraging bacteria, such as those identified in our study, to become more abundant in tumors with *TP53* mutations. The counterfactual is also possible. Similar to the *B. fragilis* toxin ETBF, which is genotoxic and initiates colon carcinogenesis in animal models [44], one or more of the tumor-associated species may induce *TP53* mutations. Notably, individuals harboring mutations in *TP53* with stage I SCC also have poorer prognosis [45], thus it will be important to determine if any of the species enriched in SCC are functionally related to reduced survival or simply biomarkers of a diminished mucosal barrier function. Whether any of these bacteria are promoting SCC tumorigenesis or inducing mutations in *TP53* is currently under investigation.

In support of the passenger hypothesis, our study indicates that smoking is associated with alterations in relative abundance of species in SCC tumors. The number one risk factor for lung cancer is tobacco exposure and is a known factor in chronic lung inflammation. Tobacco and cigarette smoke contain bacterial products (i.e. LPS) that can cause inflammation, impaired barrier function, and potentially alter the microbiome to influence lung carcinogenesis [8, 46, 47]. Additionally, tobacco leaves harbor both mold and potentially pathogenic bacteria that can be transferred in a viable form into the respiratory tract on tobacco flakes inhaled in mainstream smoke [46, 47]. Further, biologically significant quantities of bacteria are microaspirated daily in healthy individuals [48] and thus is possible for these species to accumulate in a pathogen-friendly environment but may not ultimately contribute to tumorigenesis. Nevertheless, future studies should address this issue mechanistically.

The strength of our findings includes the large number of individuals sampled in this study, use of two separate sample populations, two sets of control populations, two separate sequencing methodologies (MiSeq and PacBio), and microscopic validation (FISH) of the species in lung tumor tissue. We have also been diligent in assessing the possibility of contaminating taxa being an artifact of sample collection or sample processing by extensive quality control analysis of sequencing, sequencing across two different platforms, and microscopy. Given the low biomass of these samples, however, we were not able to completely eliminate all contaminants and acknowledge that this may skew the results. While we were able to control for antibiotic exposure in the NCI-MD study, we acknowledge a limitation of the validation study is the inability to control for antibiotic exposure in the TCGA dataset and ImA controls, as well as, significant differences in clinical features between the cancer cases and controls, which could be confounders. However, in a recent study of the microbiome of endoscopic gastric biopsies, confirmation of multiple shared bacteria in clinical samples, specifically *H. pylori*, was demonstrated using the TCGA RNA-seq data with methods similar to those presented in our study [49].

## Conclusions

With the majority of lung cancer being diagnosed at a late stage, the recent advancement in the treatment of late stage (III/IV) lung cancer with immune checkpoint inhibitors targeting PD-1, nivolumab, has resulted in a 40% reduced risk of death as compared to standard chemotherapy [50]. The response rate, however, is still not complete for these patients. Important insights into understanding the differential response rates of this new immunotherapy has suggested the composition of the lung microbiome before therapy as a key player in therapeutic effectiveness [51]. Given our results demonstrating alterations in the microbial composition in lung cancer that are histology and mutation specific, future studies should address whether the lung or nasal microbiome composition improves the stratification of patients who would be most responsive to immunotherapy. This suggestion is supported by recent animal studies demonstrating the contribution of the gut microbiome to the effectiveness of immunotherapy [52]. With these results, we foresee a new avenue for mechanistic studies to address the role of microbe-host relationship in lung cancer inflammation, response to therapy, and microbial engineering for drug delivery.

## Methods

### Sample populations and datasets

Samples used for DNA extraction, polymerase chain reaction (PCR) and sequencing were obtained from the ongoing NCI-MD study (seven hospitals participating in the greater Baltimore, MD area recruited during 1999–2012), as described previously [53], from which 398 lung cancer cases were obtained, and included both tumor and non-tumor adjacent, with 121 matched pairs. The final sample set used for analysis after sequencing, which contained 106 matched pairs after quality control, is found in Table 1. Lung tumors and paired non-tumor adjacent samples from the NCI-MD study were obtained at the time of surgery, from which a section of tumor and non-involved adjacent lung tissue from the same lung resection were flash frozen and stored at − 80 °C, with an estimated time to cold ischemia of 66 min. At the time of study entry, a detailed patient interview was conducted to obtain basic clinical information in addition to previous cancers, neoadjuvant therapies, current medications, family history of cancer, smoking history, education level, and financial status. Staging was assigned using the Cancer Staging Manual of the American Joint Committee on Cancer (AJCC) 7th edition. Preoperative antibiotics were administered for those cases recruited after 2008 and any antibiotic oral medication use was controlled for as a covariate for all statistical analysis in model testing; however, these data were not available for immediate autopsy (ImA) non-cancer samples. Controls representing non-cancerous tissue were obtained from the Lung Cancer Biorepository Research Network (*n* = 16; hospital controls). Theses samples were obtained as frozen lung specimens from individuals who had a previous positive nodule identified by PET scan and subsequently underwent tissue biopsy, which was ruled benign. The average non-operative ischemia time was 34 min (16–70 min) for these samples. Clinical information included those listed above as well as smoking history, antibiotic usage (Y/N), and disease diagnosis. Two cases had emphysema at the time of biopsy and were not used in the analyses. Immediate autopsy (ImA) samples obtained from the University of Maryland (UMD) hospital, which is part of the NCI-MD study population (*n* = 41; population controls) (Table 1). Lung tissue from ImA was received frozen from the UMD biorepository and served as the population controls for non-cancer lung tissue. Briefly, samples from ImA were obtained within minutes (< 30 min) after death and put on ice for < 30 min during dissection before cold ischemia at − 80 °C. All ImA subjects underwent extensive autopsy and were determined to be cancer-free. Demographic information included age, gender, race, and cause of death only. Non-smokers in the NCI-MD study were categorized as having smoked < 100 cigarettes or < 5 packs over a lifetime, whereas smokers were categorized as current smokers or formers smokers, who had quit for > 6 months. Sequences derived from RNA-seq of lung tumor (*n* = 1006) or non-tumor adjacent tissue (*n* = 106) were obtained from TCGA (*N* = 1112) for validation of the NCI-MD study16S rRNA gene sequencing analysis and results. Due to the fact that all RNA-seq data in TCGA were obtained using poly-A capture, any microbial data from this analysis will necessarily be biased. For this reason, we only used these data as validation of results first identified in our 16S rRNA gene sequencing analysis. Public data, including all clinical patient information (Table 1), was downloaded from the Data Matrix on the TCGA website, https://portal.gdc.cancer.gov. The raw data in the form of BAM and FastQ files were download from a secure server at CGHUB and access was applied for and approved for raw data downloads by University of California Santa Cruz, https://cghub.ucsc.edu/. The files were downloaded and stored in archived format and subsequently un-archived for analysis. The results shown here are in whole or part based upon data generated by the TCGA Research Network: https://gdc.cancer.gov.

### DNA extraction and 16S rRNA gene sequencing

DNA from lung cancer and control lung tissues was isolated according to a tissue-modified version of the standard Human Microbiome Project’s DNA isolation procedure. Genomic DNA from frozen lung tissue was extracted after tissue homogenization in Yeast Cell Lysis Buffer (Epicenter) containing lysozyme (Epicenter) by bead beating (TissueLyser II) with proteinase k (Invitrogen). DNA was purified with the Life Technologies PureLink kit according to the manufacturer’s protocol (Invitrogen). A sterile water control (MoBio) was also processed along with all frozen tissue and used as background contamination control for DNA isolation, PCR, and sequencing. Background contamination controls for tissue collection, pathology, and sequencing were also collected through routine swabs after surgery and sequenced in conjunction with tissue samples. Specifically, the NCI-MD study tissues were isolated in a laminar flow hood to minimize contamination for downstream applications, using sterile forceps and gloves. Controls for contamination points during surgical tissue collection and pathological assessment included swabs from inside of the surgical tissue collection vessel before/after, pathology cutting board before/after, pathology knife blade before/after, gloves before/after, and pathology ink bottle rim and collection tube for freezing before/after (Additional file 3). Briefly, swabs were dipped in Yeast cell Lysis buffer and area/object swabbed, then the swab was broken off into tube and frozen at − 80 °C. A negative control was also collected using 50 μL of MoBio PCR water as a mock sample (PCR_NC) and processed through DNA extraction with tissues to assess contamination from reagents, which was analyzed on three separate runs of MiSeq. The positive control was the High Even Mock Community (Broad Institute), which was also sequenced on three separate runs of MiSeq. The negative and positive control samples were spiked into four MiSeq runs at a similar concentration to that of the NCI-MD samples. To control for false grouping or batch affects, we randomized the tissue sample types (NT, T, and ImA) (with the exception of HB controls) across five separate sequencing runs of MiSeq (Additional file 4). The fifth plate consisted of duplicate samples and samples that had failed sequencing on previous runs of MiSeq.

Sequencing for the 16S rRNA gene was performed with 40 ng of sample DNA from 398 cases and 57 controls using primers for variable region V3–V5 with 16S rRNA gene sequence-specific portions based on Kozich et al. [54] with adapters for subsequent addition of standard Illumina dual indexes. PCR was performed using a Phusion DNA Polymerase High Fidelity kit (ThermoFisher). The cycling conditions were as follows: 98 °C for 2 min, then 36 cycles of 98 °C for 15 s, 60 °C for 1 min 40 s, and 74 °C for 1 min. PCR products were purified using the Agencourt AMPure XP kit according to the manufacturer’s instructions (Beckman Coulter). Second round PCR with Illumina dual-index oligos was performed using a Phusion DNA Polymerase High Fidelity kit (ThermoFisher) as following: 98 °C for 2 min, then six cycles of 98 °C for 15 s, 72 °C for 20 s, and 72 °C for 1 min. Samples were pooled and purified using Agencourt AMPure XP. Sequencing was conducted on Illumina MiSeq instrument using v3 600 cycles kit (Additional file 1: Supplemental Methods).

### Full-length 16S rDNA PCR reactions (PacBio)

Full-length 16S amplifications were performed using: 1 μL of total DNA as template; 0.25 μM of the universal 16S primers F27 and R1492 with four different sets of asymmetric barcodes at (Additional file 1: Table S9). and GoTaq Hot Start Master Mix (Promega) in a 50 μL final volume. Cycling conditions were: 94 °C, 3 min; 35 cycles of 94 °C 30 s, 54 °C 30 s, 72 °C 2 min; following by a 5 min final elongation at 72 °C. PCR products were cleaned with AxyPrep™ MagPCR (Corning Life Sciences) according to the manufacturer’s protocol and eluted in 40 μL of water. Cleaned PCR products were quantified using the Bio-Rad QX200 droplet digital PCR (Bio-Rad) and QX200 EvaGreen® Supermix with primers F357 and R534 (Additional file 1: Table S10) targeting the V3 variable region of 16S rDNA. Based on the results, amplicon libraries were normalized to the same concentration before pooling. Pooling was always performed using amplicon libraries with distinct barcodes. Multiplexing was performed with 2–4 libraries per pool.

### Pacific biosciences circular consensus sequencing

Sequencing library construction was accomplished using the Pacific Biosciences (PacBio) SMRTbell™ Template Prep Kit V1 on the normalized pooled PCR products. Sequencing was performed using the PacBio RS II platform using protocol “Procedure & Checklist - 2 kb Template Preparation and Sequencing” (part number 001–143-835- 06). DNA Polymerase Binding Kit P6 V2 was used for sequencing primer annealing and polymerase binding. SMRTbell libraries were loaded onto SMRTcells V3 at a final concentration of 0.0125 nM using the MagBead kit, as determined using the PacBio Binding Calculator software. Internal Control Complex P6 was used for all reactions to monitor sequencing performance. DNA Sequencing Reagent V4 was used for sequencing on the PacBio RS II instrument, which included MagBead loading and stage start. Movie time was 3 h for all SMRTcells. PacBio sequencing runs were set up using RS Remote PacBio software and monitored using RS Dashboard software. Sequencing performance and basic statistics were collected using SMRT® Analysis Server v2.3.0. De-multiplexing and conversion to FastQ was accomplished using the Reads of Insert (ROI) protocol in the SMRT portal v2.3 software. Only reads with a minimum of five circular passes and a predicted accuracy of 90 (PacBio score) or better were used for further analysis. Each read was labeled in the header with the number of CCS (circular consensus sequence) passes and the sample designation using a custom ruby script, followed by concatenation of all reads into a single file for subsequent filtering and clustering.

### Filtering and OTU clustering of 16S rRNA gene sequence data

Initial screening for length and quality using QIIME v 1.9.0 (qiime.org) [55]. Reads containing more than five consecutive low-quality base calls (Phred < Q20), were truncated at the beginning of the low-quality region. Due to the low quality of the majority of R2 reads (Phred < Q20 and < 150 bp length), we used the R1 reads only for this analysis. Passing sequences were required to have high-quality base calls (≥ Phred Q20) along a minimum of 75% of the read length to be included. The average Phred score per read was 34 with 88% of reads having a Phred score > 30 (Additional file 1: Supplemental Methods, Figure S1, and Table S2). After primer removal, final sequences containing ambiguous bases (Ns) or lengths < 150 bp were removed. High quality sequences were then screened for spurious PhiX contaminant using BLASTN with a word size of 16. Reads were then assessed for chimeras using USEARCH61 (de novo mode, 97% identity threshold for clustering). Non- chimeric sequences were screened for contaminant chloroplast and mitochondria using the RDP naïve Bayesian classifier, as well as non-specific human genome contaminant using Bowtie2 against the UCSC hg19 reference sequence. Finally, sequences were evaluated for residual contaminants using BLASTN searches of the GreenGenes database (v13.5). Filtered reads included those not matching any reference with at least 70% identity along 60% of their length. Exploratory assessment using BLASTN searches against the NCBI NT database indicated the majority unknown contaminant reads were amplified human genome sequence. High-quality passing sequences were subsequently clustered into operational taxonomic units using the open-reference operational taxonomic unit (OTU) picking methodology implemented within QIIME using default parameters and the GreenGenes database (99% OTUs) supplemented by reference sequences from the SILVA database (v111). Before downstream diversity analyses, the OTU table was rarefied to 5500 sequences per sample. Before diversity analysis, contaminants were removed and again OTUs table rarified to 5500 sequences per sample. Alpha diversity estimators and beta-diversity metrics were computed in QIIME with differential abundance analyses performed in R. In order to determine significant differences in beta diversity, we used the adonis function in the R package vegan to conduct PERMANOVA with Bray Curtis distance and 999 permutations in order to be able to compare across studies. All sequences from the MiSeq and PacBio datasets have been deposited at the following location: http://www.ncbi.nlm.nih.gov/bioproject/320383. See Additional file 1: Supplemental Methods for details regarding PacBio sequence processing, and Additional file 5 for complete OTU and Additional file 6 for Pathoscope results.

### TCGA RNA-seq data processing and alignment

In order to analyze all RNA-seq unmapped reads from TCGA lung cancer samples, we developed a custom metagenomic analysis pipeline using (1) MetaPhlAn2, (2) Kraken, and (3) Pathoscope [22]. First, all reads were filtered for quality using Trimmomatic (v0.32, minimum average quality > 20 over a 5-bp sliding window, minimum final length ^3^ 28 bp) and searched for potential PhiX-174 contaminant using Bowtie2. Reads passing this filter were then mapped to the comprehensive NCBI *Homo sapiens* Annotation (Release 106) using Bowtie2 to remove any human-associated reads. The resulting non-human read set was then taxonomically assigned using (1) MetaPhlAn2, (2) Kraken, and (3) Pathoscope in parallel to evaluate consistency in the resulting profiles. Assignments from each method were aggregated at higher taxonomic levels (genus and species) for downstream statistical comparisons (Additional file 1: Table S2). The results from Pathoscope and its validation in other studies lead us to use these data for the remainder of the downstream analysis.

Alpha diversity estimators and beta-diversity (Bray Curtis) metrics were computed in QIIME using genus and species level assignments with differential abundance analyses performed in R and Stata (v13). Full taxonomic assignments for each sample are provided in Additional file 5.

### Statistical analysis and classification of taxa associated with lung cancer

Statistical analysis and visualization, ANOVA and PCoA, was performed on sequencing quality metrics by population sample type (ImA, HB, NT, and T) (Additional file 1: Figure S1). Alpha- and beta-diversity metrics were computed in QIIME with differential abundance analyses performed in R and Stata (v13). Mann–Whitney tests corrected for multiple testing (Benjamini–Hochberg [FDR]) were used to conduct initial comparisons between tissue type and histological subtype (AD or SCC) followed by multivariable logistic regression controlling for multiple confounders (age, gender, race, smoking status, stage, antibiotic exposure, lung location, average Phred score, and sequencing run) (Additional file 1: Table S11). An additional logistic regression model was constructed to estimate the odds of AD versus SCC for each taxa separately (identified from the initial testing) stratified by *TP53* mutation status (wild-type versus mutated) with and interaction term between the taxa and mutation added to the model. See Additional file 1: Supplemental Methods for details of statistical modeling.

### TP53 gene sequencing and mutation analysis

Genomic DNA extracted from lung cancer tissues (*n* = 107) was submitted for *TP53*-targeted sequencing using the MiSeq Illumina platform. For mutation analysis, 46 samples were SCC. The assay was targeted at the exons and proximal splice sites. Forward and reverse primers were tailed with Illumina Adapter tags for downstream next-generation sequencing using the BioMark HD System (Fluidigm) and Access Array IFC chips and kits (Fluidigm). PCR products were indexed using an 8-mer oligo barcode. See Additional file 1: Table S3 lists sequences for primers used in the sequencing assay. Sequence results were processed and aligned to human genome and underwent QC requiring coverage > 100 reads with the variant (most single nucleotide variants [SNVs] had a read depth in the thousands) and minimum allele frequency > 10%. The 100-level cutoff for coverage allows to detect variations if the tumor fraction > ~ 20% with 95% confidence, under the assumption of a diploid genome. The 10% allele frequency cutoff is derived from that same consideration. The variants called included all common polymorphisms. Because only the tumor was sequenced, in order to score somatic mutations, those deemed to be germline were filtered out. These included SNVs present in dbSNP with high reported allele frequency (common polymorphisms). Also, SNVs in untranslated regions and introns were not considered, as their somatic status and functional implications are unclear. The presence of putative somatic exonic and splicing variants was corroborated in the TCGA and COSMIC datasets. See Additional file 1: Table S2 for details.

### Fluorescent in situ hybridization analysis of *Acidovorax*

In order to confirm the presence *Acidovorax* in lung tumor tissue, fluorescently labeled probes were created for each bacterium. Genus or species-specific bacteria probes were hybridized using tumor tissues in addition to gram stain on each. Tumor tissues from cancer cases were fixed in OCT and sectioned frozen (10 μm). Before fixation in 4% paraformaldehyde, sections were thawed at RT. Sections were washed in PBS and the probe (2 μL) was added to 90 μL FISH buffer (0.9 M NaCl, 0.02 M Tris pH 7.5, 0.01% SDS, 20% formamide). This solution was added to the section (20–100 μL) and placed in the hybridization chamber (46 °C) for 3–18 h depending on probe used. Section were washed twice (wash 1: 0.9 M NaCl, 0.02 M Tris pH 7.5, 0.01% SDS, 20% formamide; wash 2: 0.9 M NaCl, 0.02 M Tris pH 7.5, 0.01% SDS) and incubated at 48 °C for 15 min. Slides were then dried for 10 min. Before visualization, DAPI and Vectashield were added to the slides. The probe used for FISH was: *Acidovorax* (CTT TCG CTC CGT TAT CCC, 5′ modification: Alexa Fluor 532). Representative fields were imaged using Zeiss 710 and a 100X objective for the probe. In addition to two-dimensional (2D) images, Z stacks were also obtained for each bacterial probe and used to reconstruct three-dimensional (3D) images and movies using Imaris software. Quantification of *Acidovorax* probe reactivity was conducted using ten 2D fields of two patients. At least 300 cells were counted per patient. Percentage (%) of cells with perinuclear probe reactivity was quantified using ImagePro Plus 6.0 software (Additional file 1: Figure S8).

## Additional files

Additional file 1: Supplementary methods, Figures S1–S9, and Tables S1–11. (PDF 13408 kb)

Additional file 3: Background contamination swabs and relative abundance. (XLSX 145 kb)

Additional file 4: Distribution of samples across runs of 16S rRNA sequencing. (XLSX 18 kb)

Additional file 5: Clinical metadata and OTUs for NCI-MD samples. (XLSX 808 kb)

Additional file 6: Clinical metadata and taxa IDs for TCGA samples. (XLSX 17974 kb)
