# Author Correction: Interaction between the microbiome and TP53 in human lung cancer

**DOI:** 10.1186/s13059-020-01961-0

**Published:** 2020-02-20

**Authors:** K. Leigh Greathouse, James R. White, Ashely J. Vargas, Valery V. Bliskovsky, Jessica A. Beck, Natalia von Muhlinen, Eric C. Polley, Elise D. Bowman, Mohammed A. Khan, Ana I. Robles, Tomer Cooks, Bríd M. Ryan, Noah Padgett, Amiran H. Dzutsev, Giorgio Trinchieri, Marbin A. Pineda, Sven Bilke, Paul S. Meltzer, Alexis N. Hokenstad, Tricia M. Stickrod, Marina R. Walther-Antonio, Joshua P. Earl, Joshua C. Mell, Jaroslaw E. Krol, Sergey V. Balashov, Archana S. Bhat, Garth D. Ehrlich, Alex Valm, Clayton Deming, Sean Conlan, Julia Oh, Julie A. Segre, Curtis C. Harris

**Affiliations:** 1 Laboratory of Human Carcinogenesis, Center for Cancer, Research, National Cancer Institute, National Institutes of Health, 37 Convent Dr., Rm 3068A, MSC 4258, Bethesda, MD 20892-4258 USA; 2 Resphera Biosciences, Baltimore, MD 21231 USA; 3 Center for Cancer Research Genomics Core, National Cancer Institute, National Institutes of Health, Bethesda, MD 20892 USA; 4 Division of Biomedical Statistics and Informatics, Mayo Clinic, Rochester, MN 55905 USA; 5 Department of Educational Psychology, Baylor University, Waco, TX 97346 USA; 6 Laboratory of Experimental Immunology, Center for Cancer Research, National Cancer Institute, National Institutes of Health, Bethesda, MD 20892 USA; 7 Genetics Branch, Center for Cancer Research, National Cancer Institute, National Institutes of Health Bethesda, Bethesda, MD 20892 USA; 8 Department of Obstetrics and Gynecology, Mayo Clinic, Rochester, MN USA; 9 Microbiome Laboratory, Mayo Clinic, Rochester, MN 55905 USA; 10 Department of Surgery, Mayo Clinic, Rochester, MN 55905 USA; 11 Department of Microbiology and Immunology, Center for Genomic Sciences, Institute of Molecular Medicine and Infectious Disease, Drexel University College of Medicine, Philadelphia, PA 19129 USA; 12 National Human Genome Research Institute, National Institutes of Health, Bethesda, MD 20892 USA; 13 Jackson Laboratory, Framingham, CT 06032 USA; 14 Present Address: Nutrition Sciences, Baylor University, Waco, TX 97346 USA

**Author Correction to: Genome Biol**

**https://doi.org/10.1186/s13059-018-1501-6**

Following publication of the original paper [1], the authors submitted a new Additional file 5 to replace the one containing formatting issues. The updated Additional file 5 is published in this correction.

## Supplementary information

**Additional file 5.** Clinical metadata and OTUs for NCI-MD samples.
