# Supplementary material for: Interaction between the microbiome and TP53 in human lung cancer
Source: Genome Biol. 2018 Aug 24;19:123. doi: 10.1186/s13059-018-1501-6 (PMC6109311; doi:10.1186/s13059-018-1501-6)
Supplement: Supplementary file 2 — Supplementary methods, Figures S1–S9, and Tables S1–11. (PDF 13408 kb) [file 13059_2018_1501_MOESM1_ESM.pdf]

SUPPLEMENTAL METHODS

PacBio: Filtering, clustering, and taxonomic classification of CCS reads

To remove reads that were off-target or likely contained errors, we used a set of filters to exclude CCS reads that: (a) were less than 500 or more than 2000 base pairs; (b) mapped to the human genome (GRCh37) with BWA v0.7.10-r789 mem using PacBio settings (8); or (c) did not match both forward and reverse PCR primers using USEARCH v8.1.1861_i86linux6 (9). Primer sequences were then trimmed off, and reads were oriented 5' to 3' with respect to 16S rRNA transcription.

To cluster reads into operational taxonomic units (OTU), we performed our original binning using only those CCS reads with a cumulative expected error of less than 1 (*i.e.* the sum of phred-scaled quality scores across the ~1.5 kb predicts that the sequence would contain less than 1 error). Using the uparse suite of tools, these CCS reads were de-replicated and clustered into OTUs defined at a 97% sequence identity threshold. These centroid OTU sequences were filtered for chimeras with uchime (10) using default parameters and the RDP gold database (11). Centroid OTUs were classified using utax with a curated full-length 16S rRNA database from the NCBI, which provided species-level taxonomic IDs for most sequences, and confidence values at each taxonomic level were also provided to the species level. A detailed description of how the database used for the classifier was built can be found at https://github.com/bhatarchanas/lineanator. To count the abundance of each centroid OTU in each sample, all primer-matched reads were mapped back to the centroid OTU sequences using usearch global.

MiSeq: Sequence quality analysis by tissue sample population

Due to our exclusion of the R2 reads, use of multiple tissues samples from disparate population, multiple sequencing runs, and high PCR cycling conditions we conducted a thorough analysis of sequencing quality metrics stratified by study population to examine their influence on our results. For this analysis we calculated the average Phred score per read (AvgPhred) and percent of reads with a Phred score >25%, 30% or 35% (Additional file 1: Table S2). Using these categories, we visualized distribution of each metric by sample population and by *Acidovorax* abundance (Additional file 1: Fig. S1). In addition, we performed the ANOVA test to determine if there was significant difference in average Phred score by sample type, which was significant (DF=3; Sum Sq=16.0; Mean Sq=5.34; F-val=12.37; p-val<0.001) across tissue types. Post-hoc Tukey tests demonstrated that significant difference was attributed to the ImA samples as compared to the other tissue types. While there was difference in Phred quality between ImA and HB, there was no difference in alpha or beta diversity between these samples; though we cannot rule out a potential effect on these results. Given this information, we controlled for Phred scores in our regression analyses (Additional file 1: Table S11).

Identification of taxa associated with lung cancer histological subtype*:* Mann-Whitney tests corrected for multiple testing (Benjamini–Hochberg (FDR)) were used to conduct initial comparisons between tissue type (tumor, non-tumor, hospital biopsy or immediate autopsy) and histological subtype (AD or SCC). Unconditional logistic regression was conducted and odds ratios (ORs) were calculated for each taxa present in AD or SCC tumors from 16S rRNA gene sequencing and RNA-seq (TCGA). Taxa found to be significantly differentially abundant between AD and SCC tumors (p-value

<0.05) were put into an adjusted model to control for age, gender, race, smoking status, stage, and lung location, and p-values were corrected for multiple hypothesis testing using FDR adjustment. Given the association between Phred scores and histological subtypes (SCC and AD), we added this additional covariate to our previous model, and after adjustment we saw very little change in the OR, which remained significant (Additional file 1: Table S11). In a separate multivariable model, we also adjusted for time since smoking cessation among former smokers (1-9, 10-20 or ≥20 years). Due to fewer AD and SCC tumors in the NCI-MD study as compared to the TCGA study, we only kept those genera or species in common between the two studies that had a FDR-corrected p-value <0.05 in TCGA study. The best-fit model for each analysis was chosen based on Akaike information criterion (AIC), and in addition to those listed above included antibiotics usage, family history of cancer, previous cancer history and treatments (chemotherapy and radiation). However, antibiotic exposure was not available in the TCGA dataset. For identification of taxa differentially abundant in tumors between smokers and non-smokers we used the algorithm LEfSe (16), and then stratified by smoking status, never, former or current, and conducted Kruskal-Wallis tests and Dunn's multiple comparisons test (alpha <0.05) to identify statistical differences between groups. To determine if an interaction existed between SCC-associated taxa and *TP53* mutation status, a logistic regression model was constructed to estimate the odds of AD vs SCC for each taxa stratified by mutation status with and interaction term between the taxa and mutation added to the model. In adjusted logistic regression models for both NCI-MD and TCGA data sets, the interaction between SCC-associated taxa and mutation status was significant (p-value<0.05).

Speciation of genera from 16S rRNA gene sequencing analysis*:* To perform high-resolution characterization of OTUs assigned to *Variovorax* and *Streptococcus*, all sequences within these OTUs were evaluated using Resphera Insight (Baltimore, MD) (17). The Insight protocol utilizes a manually curated reference database with over 11,000 unique species and a novel hybrid local/global alignment strategy to perform ultra-high-resolution taxonomic assignment of partial 16S rRNA gene sequence fragments.

SUPPLEMENTAL FIGURES

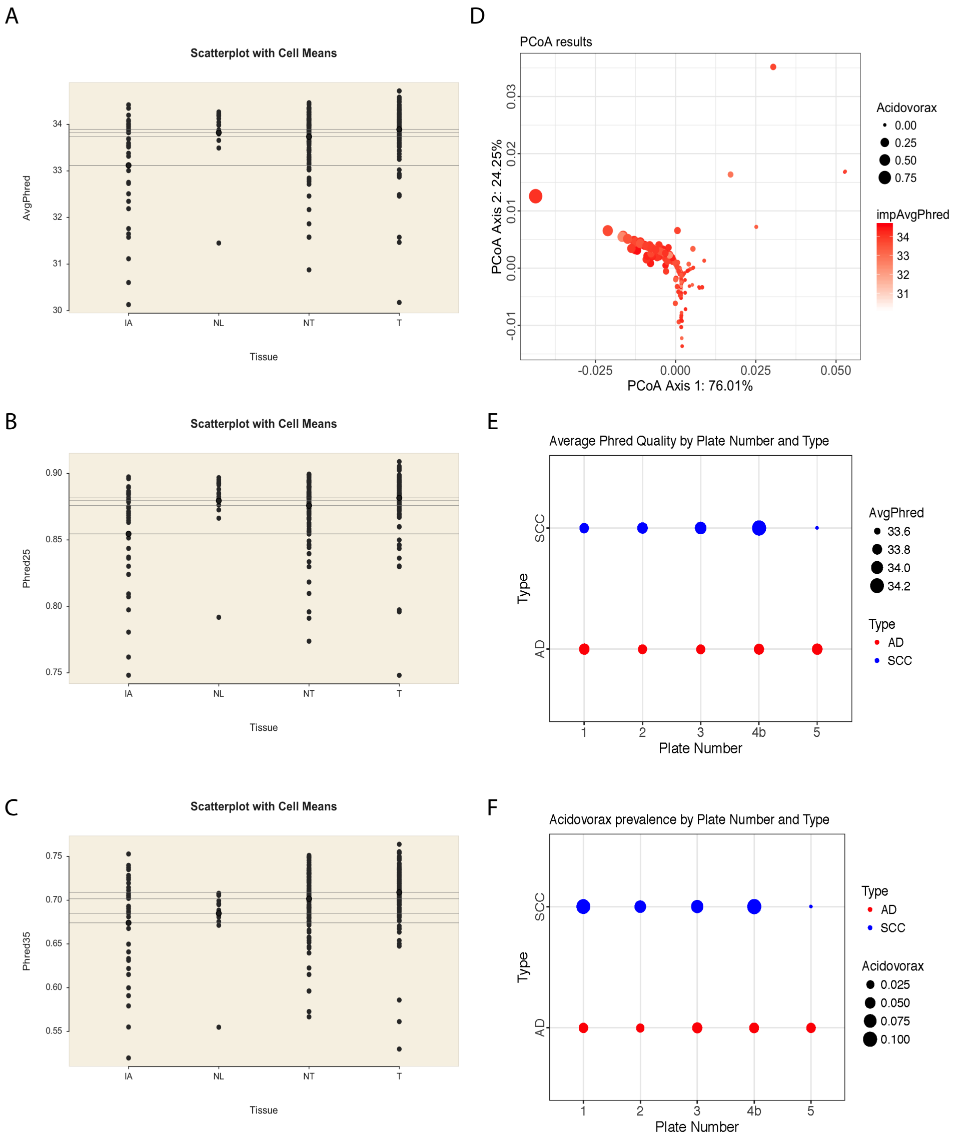

Fig.S1: Sequencing quality analysis. Phred sequencing quality score distribution by sample tissue type using A) average Phred score, B) percent of reads with Phred score >25 Phred, and C) percent of reads with Phred score >35. D) PCoA plot of the Bray Curtis distances between subjects, with Phred average per read scaled by red color intensity and *Acidovorax* relative abundance by scaled by circle size. Average Phred score E) and *Acidovorax* relative abundance F) by histological subtype, AD and SCC, divided by plate number.

AI=immediate autopy, NL=hospital biopsy, NT=non-tumor adjacent, T=tumor.

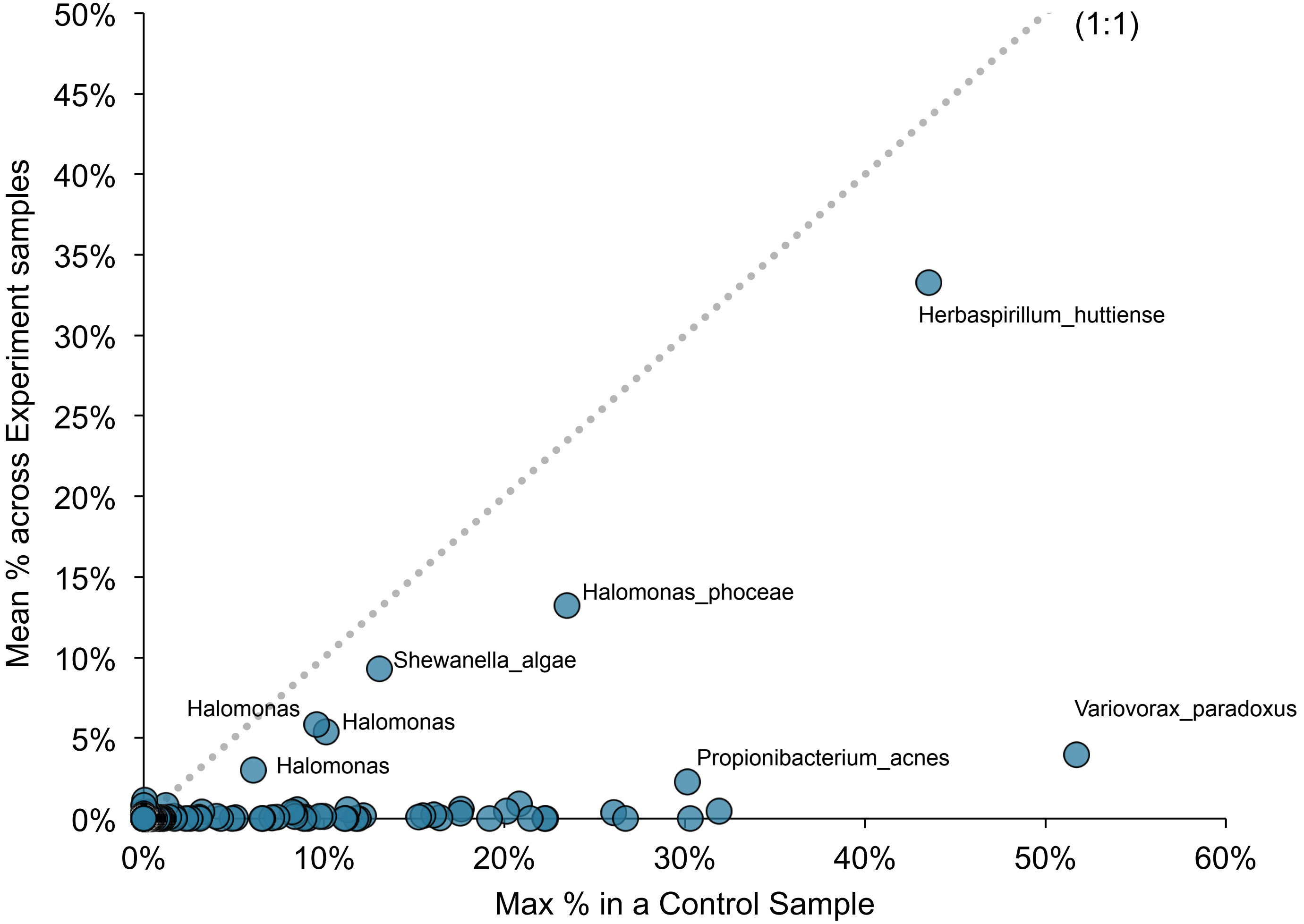

Fig. S2. Plot comparing relative abundance of microbes present in contamination control and experimental tissue samples. Taxonomic assignments were based on open-reference OTU picking with QIIME.

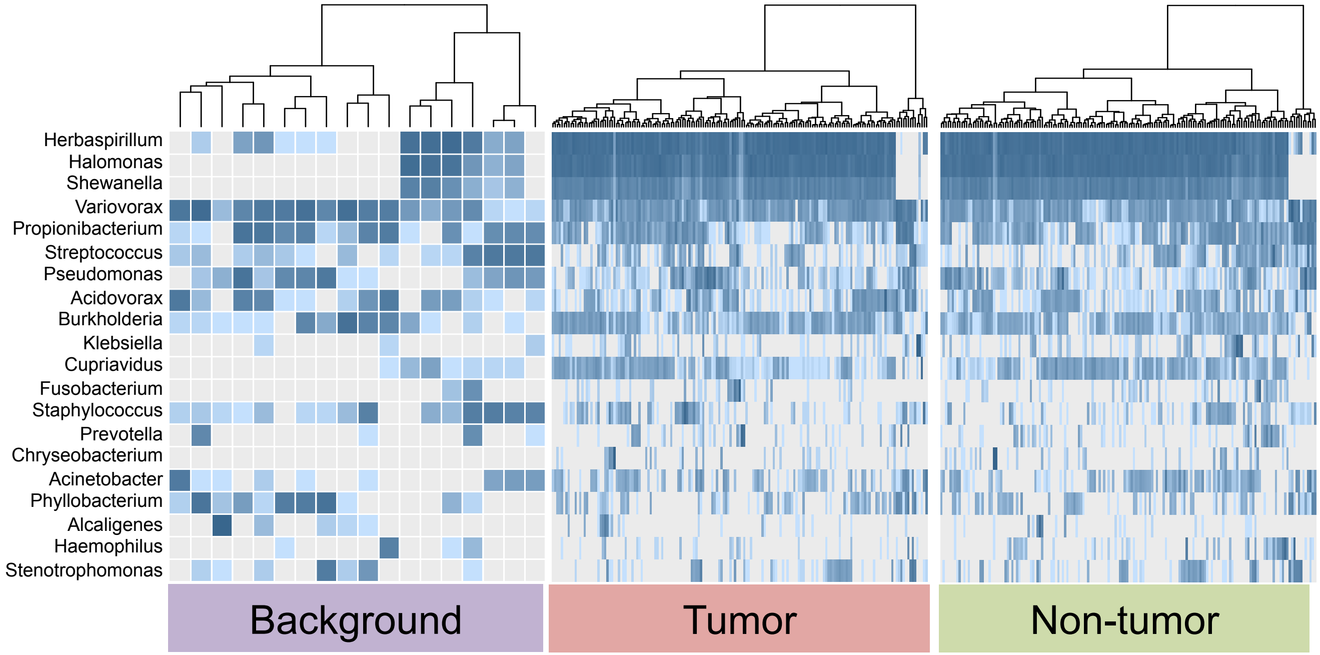

Fig. S3. Hierarchal clustering of microbial relative abundance in contamination background controls and tissues samples by tissue type

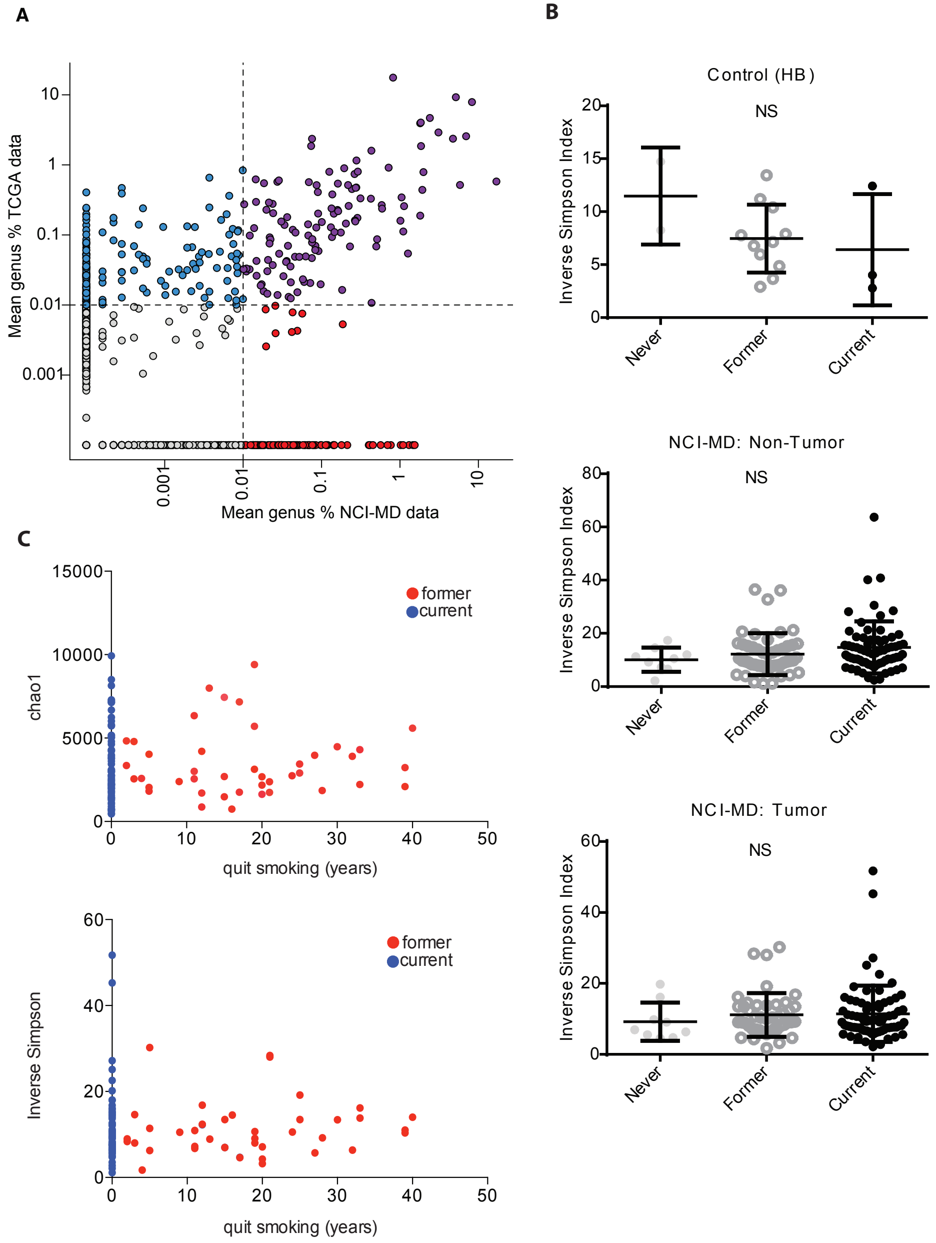

FigS4. Diversity between studies and by smoking exposure. A) Shared genera between the 16S rRNA gene sequencing and metatranscriptomic (TCGA) data set, thresholded at 0.01 % of mean genus abundance for all tumor and non-tumor samples. B) Comparison of diversity (Inverse Simpson) stratified by smoking status among controls (HB), non-tumor and tumor tissue in the NCI-MD study. C) Association between richness (chao1) or diversity (Inverse Simpson) and time since quitting smoking between former and current smokers in the NCI-MD study.

(HB=hospital biopsy)

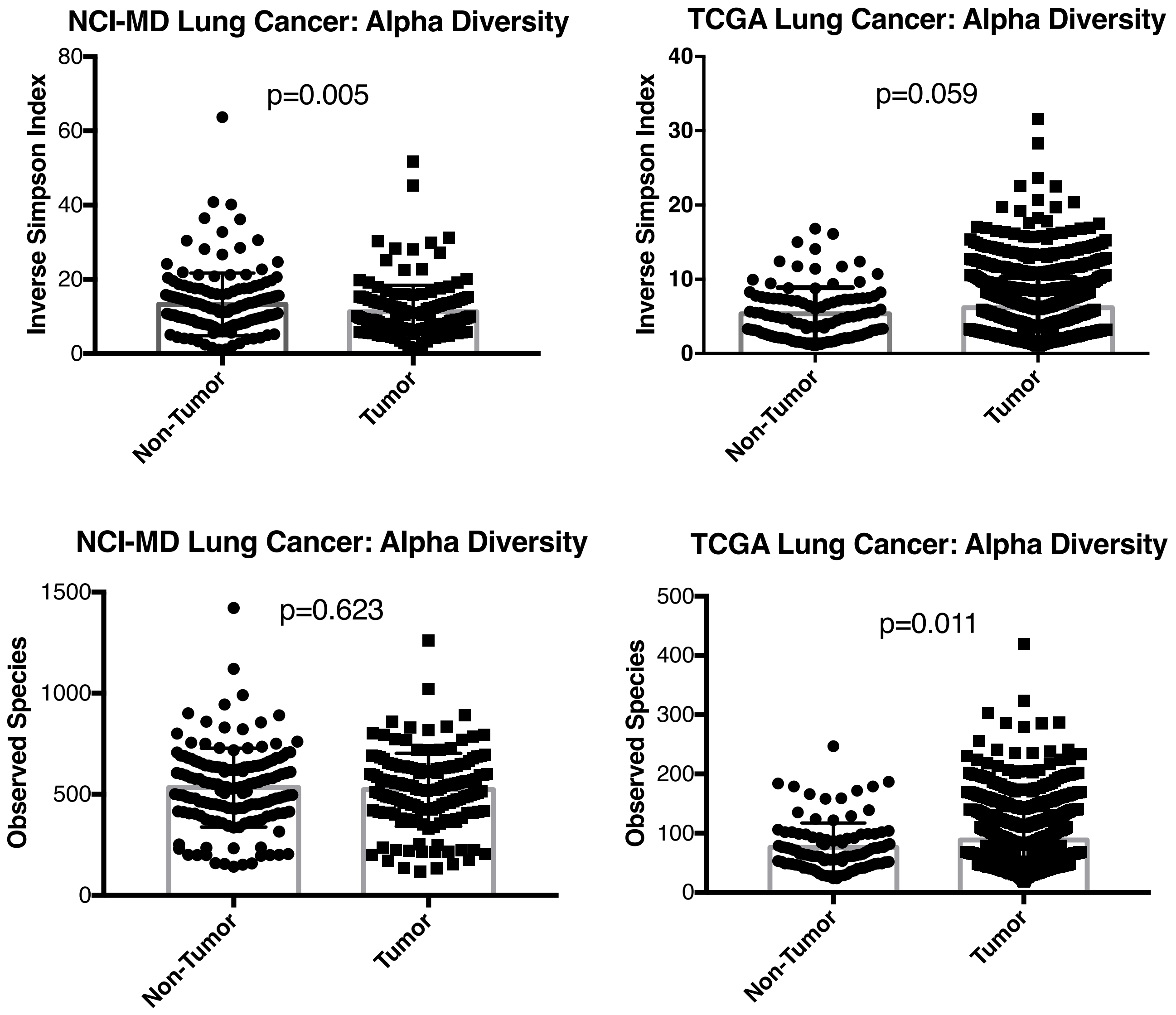

Fig. S5: Diversity analysis between tumor and non-tumortissue. Inverse Simpson diversity A-B) and observed species C-D) between tumor and non-tumor tissue in the NCI-MD study and the TCGA study, with significance determined by Welches t-test.

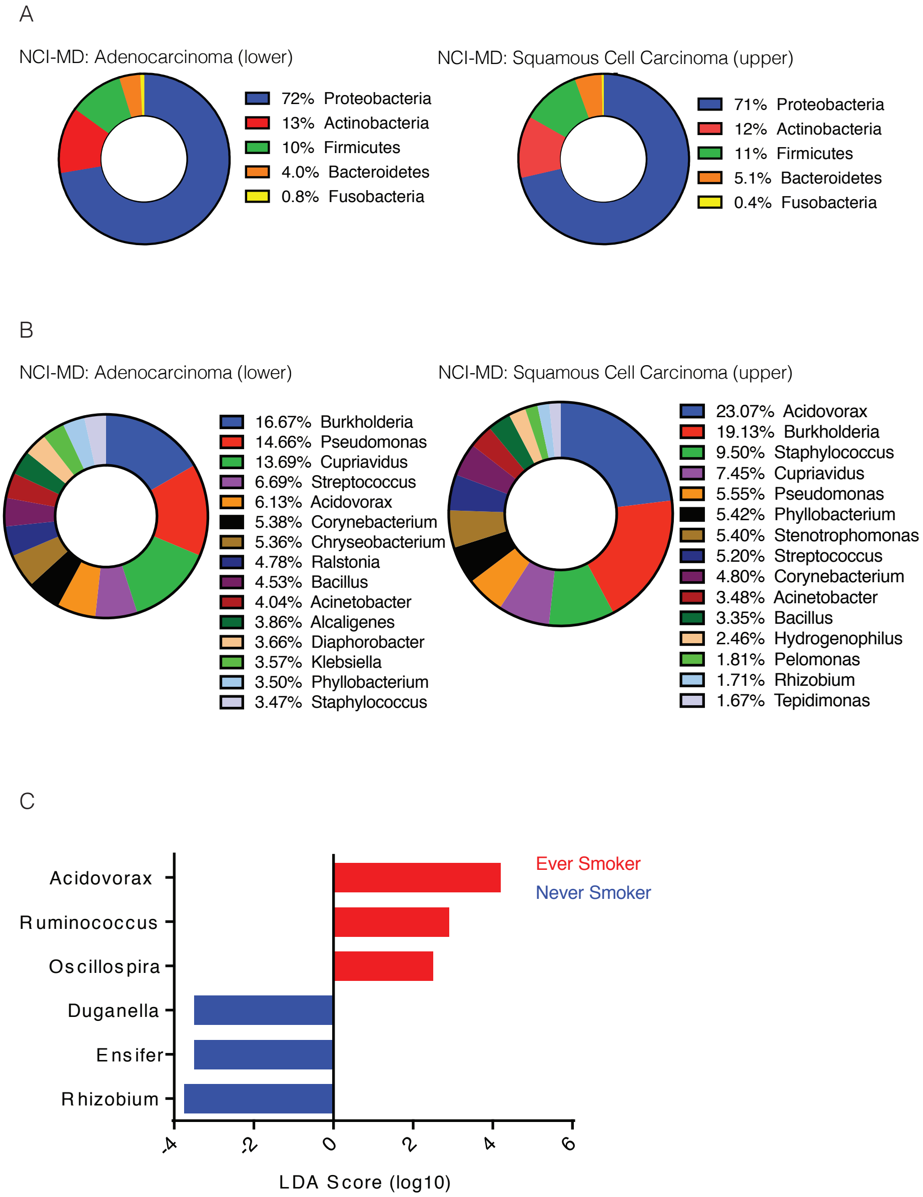

Fig. S6. Comparison of taxonomic abundance by lung location and between never smokers and ever-smokers (current and former smokers) in tumor tissue. A) Top most abundant phyla from the NCI-MD data set in adenocarcinomas from lower lung (left) or squamous cell carcinoma from upper lung (right). B) Top 15 most abundant genera (relative abundance) in the NCI-MD data set in adenocarcinomas from lower lung (left) or squamous cell carcinoma from upper lung (right). C) Using linear discriminate analysis (LDA) coupled with effect size, we identified *Acidovorax* as the most differentially abundant taxon in tumor tissue between never smokers and ever smokers in samples from the NCI-MD data set.

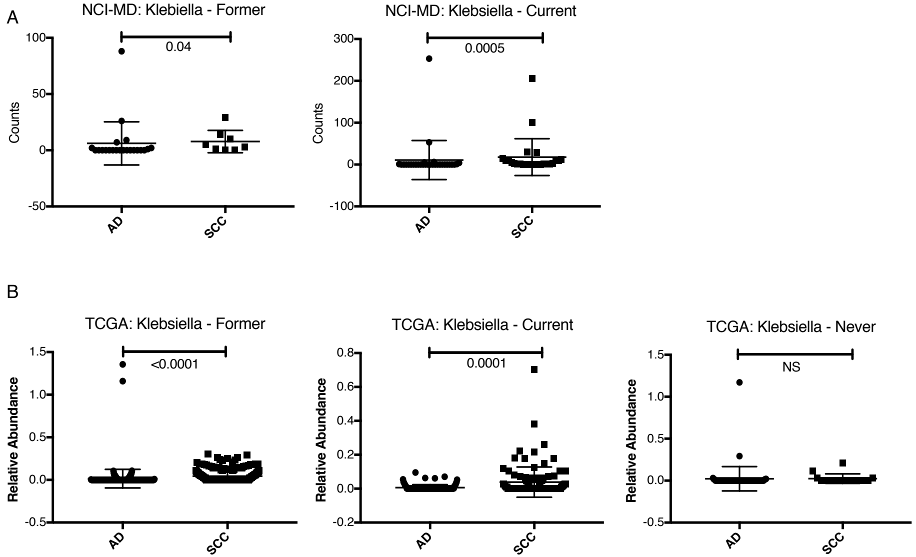

Fig.S7. Relative abundance of *Klebsiella* stratified by smoking status and histological subtype. A) Relative abundance of *Klebsiella* stratified by smoking status and histological subtype in NCI-MD study, *Klebsiella* was not identified in never smokers . B) Relative abundance of *Klebsiella* stratified by smoking status and histological subtype in TCGA study. Test of significance are Mann-Whitney.

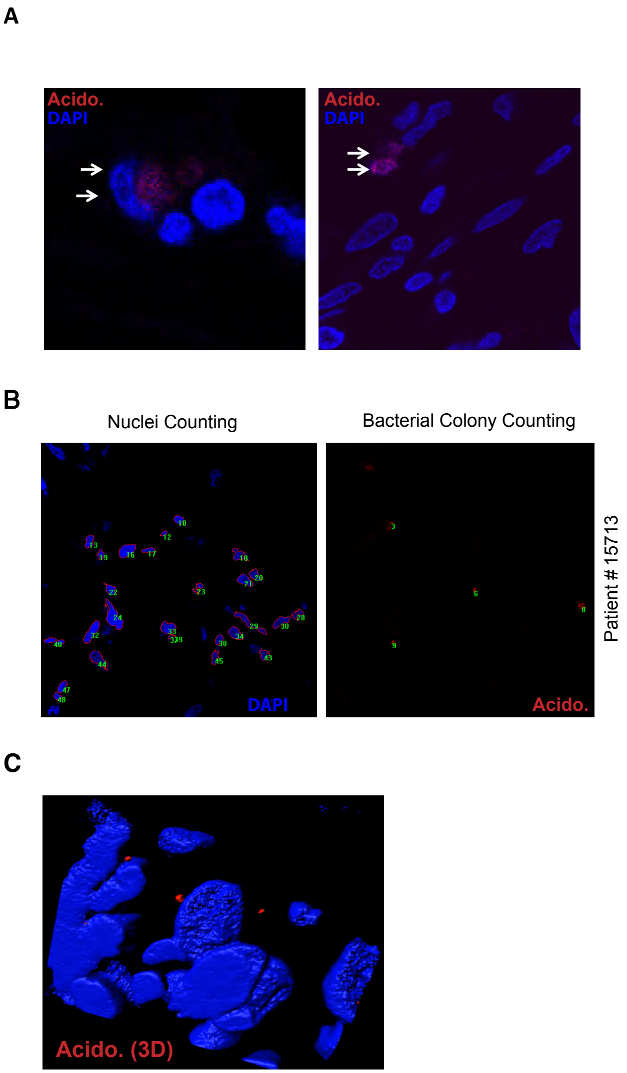

Fig. S8: Comparison of putative species in NCI-MD study from speciation of 16S rRNA gene reads to TCGA study and histological quantification. A) Representative images of *Acidovorax* in a representative patient. F) Representative images of tumor tissue ((63X) quantified using 10 fields for each section of a representative patient positive for *Acidovorax* probe reactivity. A total of at least 300 cells were counted; nuclei counting using blue DAPI and bacterial colony counting using red bacteria specific probe. G) Representative image of a 3D movie (*Video S1*) of reconstructed Z stack images of *Acidovorax* (bottom panel) positive tumor tissues.

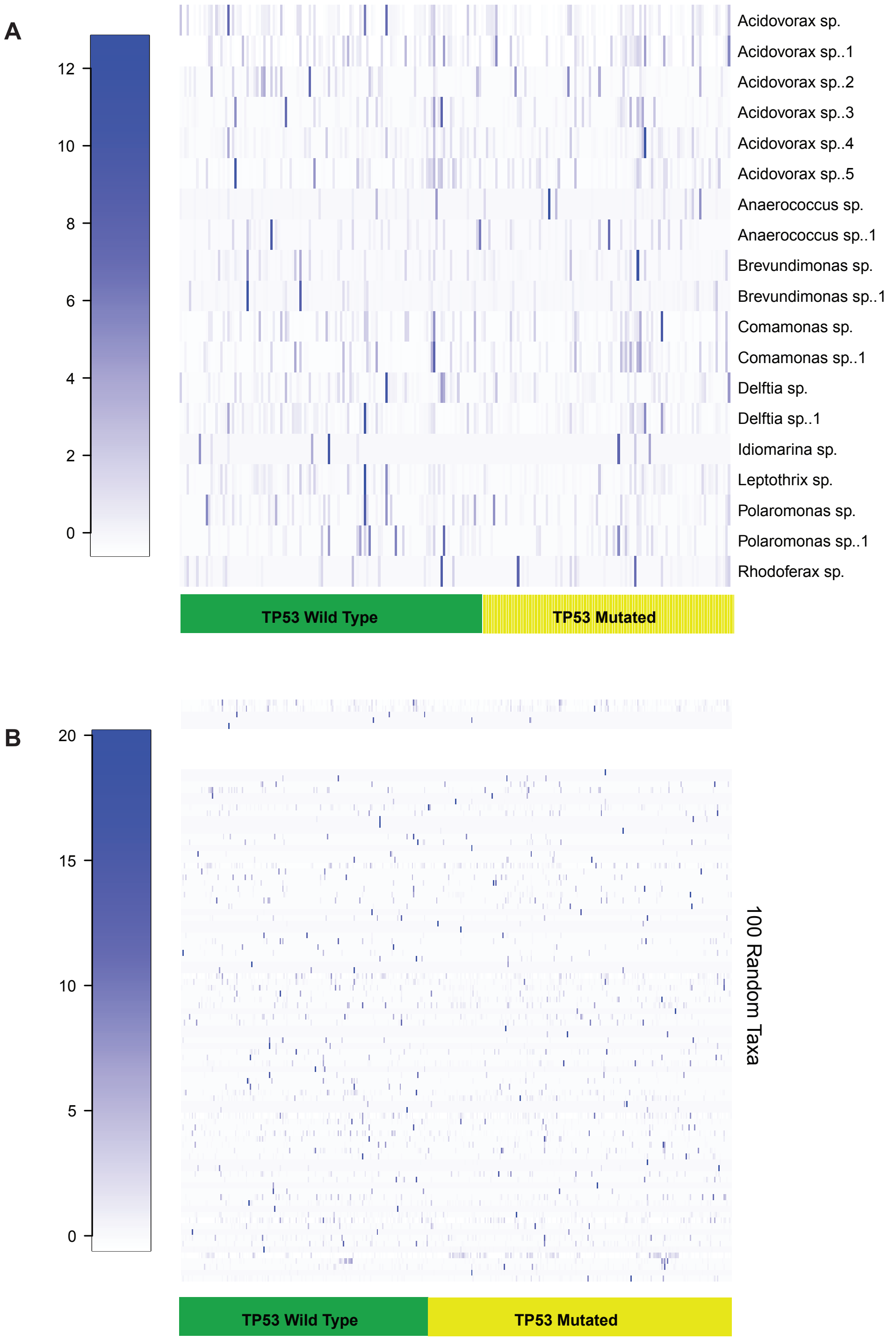

Fig.S9 Heat maps showing relative abundance of taxa that differentiate AD and SCC or randomly chosen taxa comparing mutation status in all AD tumors. A) Heat map of relative abundance of taxa demonstrating no pattern of enrichment in AD tumors. B) Heat map of relative abundance of 100 randomly chosen taxa does not demonstrate pattern of enrichment in taxa in all tumors.

SUPPLEMENTARY TABLES

<table>
<tr><th>Contaminants removed</th><th>Mann-Whitney P- vals <= 0.05</th><th>Removal order</th><th>Contaminant List</th></tr>
<tr><td>None</td><td>20</td><td>0</td><td>None</td></tr>
<tr><td>top1</td><td>18</td><td>1</td><td>Unassigned;Other;Other;Other;Other;Other</td></tr>
<tr><td>top2</td><td>14</td><td>2</td><td>k__Bacteria;Other;Other;Other;Other;Other</td></tr>
<tr><td>top3</td><td>20</td><td>3</td><td>k__Bacteria;p__Proteobacteria;c__Betaproteobacteria;o__Burkholderiales;f__Oxalobacteraceae;g__Herbaspirillum</td></tr>
<tr><td>top4</td><td>20</td><td>4</td><td>k__Bacteria;p__Proteobacteria;c__Gammaproteobacteria;o__Oceanospirillales;f__Halomonadaceae;g__Halomonas</td></tr>
<tr><td>top5</td><td>37</td><td>5</td><td>k__Bacteria;p__Proteobacteria;c__Gammaproteobacteria;o__Alteromonadales;f__Shewanellaceae;g__Shewanella</td></tr>
<tr><td>top6</td><td>30</td><td>6</td><td>k__Bacteria;p__Actinobacteria;c__Actinobacteria;o__Actinomycetales;f__Propionibacteriaceae;g__Propionibacterium</td></tr>
<tr><td>top7</td><td>26</td><td>7</td><td>k__Bacteria;p__Proteobacteria;c__Betaproteobacteria;o__Burkholderiales;f__Comamonadaceae;g__Variovorax</td></tr>
<tr><td>top8</td><td>23</td><td>8</td><td>k__Bacteria;p__Proteobacteria;c__Betaproteobacteria;o__Burkholderiales;f__Comamonadaceae;g__Acidovorax</td></tr>
<tr><td>top9</td><td>32</td><td>9</td><td>k__Bacteria;p__Proteobacteria;c__Betaproteobacteria;o__Burkholderiales;f__Burkholderiaceae;g__Burkholderia</td></tr>
<tr><td>top10</td><td>31</td><td>10</td><td>k__Bacteria;p__Proteobacteria;c__Gammaproteobacteria;o__Pseudomonadales;f__Pseudomonadaceae;g__Pseudomonas</td></tr>
<tr><td>top11</td><td>31</td><td>11</td><td>k__Bacteria;p__Proteobacteria;c__Betaproteobacteria;o__Burkholderiales;f__Oxalobacteraceae;g__Cupriavidus</td></tr>
<tr><td>top12</td><td>29</td><td>12</td><td>k__Bacteria;p__Firmicutes;c__Bacilli;o__Bacillales;f__Bacillaceae;g__Bacillus</td></tr>
<tr><td>top13</td><td>28</td><td>13</td><td>k__Bacteria;p__Proteobacteria;c__Betaproteobacteria;o__Burkholderiales;f__Oxalobacteraceae</td></tr>
<tr><td>top14</td><td>28</td><td>14</td><td>k__Bacteria;p__Proteobacteria;c__Betaproteobacteria;o__Burkholderiales;f__Comamonadaceae</td></tr>
<tr><td>top15</td><td>23</td><td>15</td><td>k__Bacteria;p__Proteobacteria;c__Gammaproteobacteria;o__Oceanospirillales;f__Halomonadaceae</td></tr>
<tr><td>top16</td><td>17</td><td>16</td><td>g__(Afipia|Aquabacteriume|Asticcacaulis|Aurantimonas|Beijerinckia|Bosea|Bradyrhizobium|Brevundimonas|Caulobacter|Craurococcus|Devosia|Hoefleae|Mesorhizobium|Methylobacterium|Novosphingobium|Ochrobactrum|Paracoccus|Pedomicrobium|Phyllobacteriume|Rhizobium|Roseomonas|Sphingobium|Sphingomonas|Sphingopyxis|Acidovorax|Azoarcuse|Azospira|Burkholderia|Comamonas|Cupriavidus|Curvibacter|Delftia|Duganella|Herbaspirillum|Janthinobacteriu|Kingella|Leptothrixa|Limnobactere|Massiliac|Methylophilus|Methyloversatilise|Oxalobacter|Pelomonas|Polaromonas|Ralstonia|Schlegelella|Sulfuritalea|Undibacteriume|Variovorax|Acinetobactera|Enhydrobacter|Enterobacter|Escherichia|Nevskia|Pseudomonas|Pseudoxanthomonas|Psychrobacter|Stenotrophomonas|Xanthomonas|Aeromicrobium|Arthrobacter|Beutenbergia|Brevibacterium|Corynebacterium|Curtobacterium|Dietzia|Geodermatophilus|Janibacter|Kocuria|Microbacterium|Micrococcus|Microlunatus|Patulibacter|Propionibacteriume|Rhodococcus|Tsukamurella|Abiotrophia|Bacillus|Brevibacillus|Brochothrix|Facklamia|Paenibacillus|Streptococcus|Chryseobacterium|Dyadobacter|Flavobacterium|Hydrotalea|Niastella|Olivibacter|Pedobacter|Wautersiella|Deinococcus)</td></tr>
</table>

"top16" includes all of the genera identified in Salter et al. 2014 Table 1

Table S1: Systematic removal process of putative contaminants. The number of Mann-Whiteny p-values < 0.05 comparing paired tumor normal samples after stepwise removal of top i^{th} taxa for the top 15 putative contaminants ($i^{th}{}_{1\text{-}15}$). Additional analysis included the removal of all environmental contaminants (top 16) identified in Salter et al. 2014. After removal of the top 5 putative contaminants, the largest increase in p-values (37) is observed.

Table S2: Results of sequencing from 16S rRNA gene sequencing data set and reads counts from each filtering step in the quality control pipeline.

Sequencing run	Sample	Phred (AVG)	Phred (Min)	Phred (Max)	% Phred >30	Passed qiime filtering	Primer removal failures	Primer removal success	PhiX hits	Total chimeras	Total known contaminant	Total unknown contaminant	Final clean seqs
1	Total					11635283	21251	11614032	265	628946	786960	10234	10187627
	Average	33.81959	31.46605	34.33492	82.55162	130733.5	238.8	130494.7	3	7066.8	8842.2	115	114467.7
2	Total					12243607	8953	12234654	637	512071	1157373	11974	10552599
	Average	33.77584	30.17505	34.48731	82.135	128880.1	94.2	128785.8	6.7	5390.2	12182.9	126	111080
3	Total					11211910	57005	11154905	640	318230	983722	4469	9847844
	Average	33.86385	30.87568	34.7178	82.62368	118020.1	600.1	117420.1	6.7	3349.8	10355	47	103661.5
4	Total					5407498	2	5407496	259	47873	2185681	16572	3157111
	Average	33.84671	31.45097	34.48626	83.25612	142302.6	0.1	142302.5	6.8	1259.8	57517.9	436.1	83081.9

TCGA Sequencing Results

	Sequences Passing Contaminant Screen	Sequences Annotated by Pathoscope	Pathoscope Alignments	Metaphlan Alignments	Kraken Alignments
Average	943668.914	36216	82	208.81205	4477.54635
Median	615311.5	31116	68.5	99	2030

Table S3. List of primers and targets for TP53 exon sequencing

Forward Primer Name	Sequence Forward	Reverse Primer Name	Sequence Reverse	Target Region	Size
TP53_Ex2.1F	tcccacaggtctctgctagg	TP53_Ex2.1R	tggaagtgtctcatgctgga	chr17:7579765+7579981	217
TP53_Ex3.1F	aaaagagcagtcagaggacc	TP53_Ex3.1R	ccatgggactgactttctgc	chr17:7579604+7579754	151
TP53_Ex4.1aF	caggcattgaagtctcatgg	TP53_Ex4.1aR	gaagacccaggtccagatga	chr17:7579267+7579521	255
TP53_Ex4.1bF	ttctgggaagggacagaaga	TP53_Ex4.1bR	cctggtcctctgactgctct	chr17:7579385+7579626	242
TP53_Ex5.1F	gccctgtcgtctctccag	TP53_Ex5.1R	gccctgactttcaactctgtct	chr17:7578339+7578593	255
TP53_Ex6.1F	cttaacccctcctcccagag	TP53_Ex6.1R	ctgctcagatagcgatggtg	chr17:7578137+7578387	251
TP53_Ex7.1F	gggtcagaggcaagcagag	TP53_Ex7.1R	ttgggcctgtgttatctcct	chr17:7577433+7577630	198
TP53_Ex8.1F	gcttcttgtcctgcttgctt	TP53_Ex8.1R	ggtggttgggagtagatgga	chr17:7576997+7577251	255
TP53_Ex9.1F	ccccaattgcaggtaaaaca	TP53_Ex9.1R	ggagaccaagggtgcagtta	chr17:7576759+7576990	232
TP53_AltSpl.1F	aggctaagctatgatgttcctt	TP53_AltSpl.1R	caatggctcctggttgtagc	chr17:7576470+7576724	255
TP53_Ex10.1F	gaaggcaggatgagaatgga	TP53_Ex10.1R	acttctccccctcctctgtt	chr17:7573842+7574060	219
TP53_Ex11.1F	caagggttcaaagacccaaa	TP53_Ex11.1R	tgtcatctctcctccctgct	chr17:7572838+7573042	205
TP53_Ex2.2F	tccaatggatccactcacag	TP53_Ex2.2R	atccccacttttcctcttgc	chr17:7579821+7579962	142
TP53_Ex3.2F	ggtgaaaagagcagtcagagg	TP53_Ex3.2R	cgaaaattccatgggactga	chr17:7579600+7579762	163
TP53_Ex4.2aF	gcattgaagtctcatggaagc	TP53_Ex4.2aR	gaagacccaggtccagatga	chr17:7579270+7579521	252
TP53_Ex4.2bF	ctgggaagggacagaagatg	TP53_Ex4.2bR	gacctggtcctctgactgct	chr17:7579387+7579628	242
TP53_Ex5.2F	aaccagccctgtcgtctct	TP53_Ex5.2R	tgtctccttcctcttcctacag	chr17:7578334+7578576	243
TP53_Ex6.2F	aaccacccttaacccctcct	TP53_Ex6.2R	caggcctctgattcctcact	chr17:7578130+7578321	192
TP53_Ex7.2F	tggaagaaatcggtaagaggtg	TP53_Ex7.2R	cttgggcctgtgttatctcc	chr17:7577404+7577631	228
TP53_Ex8.2F	accgcttcttgtcctgctt	TP53_Ex8.2R	gggagtagatggagcctggt	chr17:7576994+7577244	251
TP53_Ex9.2F	gaaaacggcattttgagtgtt	TP53_Ex9.2R	aagggtgcagttatgcctca	chr17:7576787+7576983	197
TP53_AltSpl.2F	ggctaagctatgatgttcctt	TP53_AltSpl.2R	acaatggctcctggttgtag	chr17:7576471+7576725	255
TP53_Ex10.2F	ggaatcctatggctttccaa	TP53_Ex10.2R	ttctccccctcctctgttg	chr17:7573859+7574058	200
TP53_Ex11.2F	cagtggggaacaagaagtgg	TP53_Ex11.2R	tcatctctcctccctgcttc	chr17:7572901+7573040	140

Table S4: Results from PacBio sequencing using full length 16S rRNA primers on 57 tumor and non-tumor adjacent DNA samples from the NCI-MD study.

genus	Acidovorax	
species	PacBio* Acidovorax_temperans	MiSeq (genus only)
Present	4	51
Absent	53	6

*Total number of samples in PacBio sequencing = 57

Table S5. Association between genus level taxonomic abundance and odds of squamous cell carcinoma in NCI-MD tumor tissue

Taxa	OR (95% CI)*	P	FDR
g_Tepidimonas	1.95(0.68-3.22)	0.0026	0.23
g_Brevundimonas	2.64(0.77-4.51)	0.0057	0.23
g_Acidovorax	1.53(0.38-2.68)	0.0093	0.23
g_Klebsiella	1.38(0.17-2.59)	0.025	0.46
g_Anaerococcus	1.96(0.18-3.74)	0.031	0.46
g_Rhodoferax	1.51(0.03-3.06)	0.054	0.46

*Adjusted for age, gender, race, location, smoking, stage, packyears n=120

Table S6. Association between species level taxonomic abundance and odds of squmaous cell carcinoma in tumor tissue in TCGA tumors

Taxa	OR (95% CI)*	P	FDR
Klebsiella_oxytoca_E718	5.25(3.781-7.31)	4.3E-23	2.76E-20
Acidovorax_sp._KKS102	4.85(3.52-6.753)	2.1E-21	6.75E-19
Acidovorax_avenae_ATCC_19860	3.38(2.48-4.61)	1.1E-14	6.43E-13
Acidovorax_sp._JS42	2.61(1.95-3.52)	2.2E-10	4.49E-09
Rhodoferax_ferrireducens_T118	2.58(1.93-3.49)	2.7E-10	5.34E-09
Acidovorax_citrulli_AAC00-1	2.5(1.85-3.38)	1.6E-09	2.94E-08
Acidovorax_ebreus_TPSY	2.5(1.85-3.38)	2.1E-09	3.72E-08
Klebsiella_pneumoniae_KCTC_2242	2.48(1.84-3.35)	2.8E-09	4.83E-08
Acidovorax_delafieldii_2AN	2.31(1.71-3.12)	2.8E-08	4.02E-07
Anaerococcus_hydrogenalis_DSM_74	0.67(0.49-0.89)	0.0077	0.03453578

*Adjusted for age, gender, race, location, smoking and stage n=846

Table S7: Summary statistics for relative abundance of taxa in NCI Data

Taxa	Mean[1]	SD	Skew	Kurtosis	Min	Q1	Mdn	Q3	Max	%-Freq.[2]
Acidovorax	0.0474	0.09	3.90	27.86	0.00	0.00	0.00	0.07	0.91	0.91
Brevundimonas	0.0027	0.01	7.88	70.73	0.00	0.00	0.00	0.00	0.17	0.21
Comamonas	0.0027	0.01	5.81	36.57	0.00	0.00	0.00	0.00	0.12	0.45
Tepidimonas	0.0041	0.02	5.26	31.20	0.00	0.00	0.00	0.00	0.16	0.36
Rhodoferax	0.0005	0.01	17.55	314.10	0.00	0.00	0.00	0.00	0.12	0.28
Klebsiella	0.0146	0.09	8.99	85.40	0.00	0.00	0.00	0.00	0.99	0.52
Leptothrix	0.0001	0.00	4.28	26.62	0.00	0.00	0.00	0.00	0.00	0.41
Polaromonas	0.0001	0.00	3.22	12.47	0.00	0.00	0.00	0.00	0.00	0.25
Anaerococcus	0.0008	0.01	11.17	133.43	0.00	0.00	0.00	0.00	0.10	0.05

1 Mean relative abundance

2 Percent frequency among all samples

Table S8: Summary statistics for relative abundance of taxa in TCGI Data

Taxa	Mean	SD	Skew	Kurtosis	Min	Q1	Mdn	Q3	Max	%-Freq.
Acidovorax	1.11	2.37	3.26	10.38	0.00	0.14	0.30	0.63	15.02	0.99
taxid 232721: Acidovorax sp JS42	0.32	0.61	2.88	8.07	0.00	0.01	0.09	0.26	3.56	0.82
taxid 358220: Acidovorax sp KKS1	0.32	0.69	3.38	11.58	0.00	0.01	0.07	0.24	4.50	0.88
taxid 397945: Acidovorax citrulli	0.06	0.16	3.29	10.69	0.00	0.00	0.00	0.02	1.00	0.55
taxid 398578: Delftia acidovorans	0.39	0.90	3.17	10.25	0.00	0.00	0.04	0.24	5.77	0.79
taxid 535289: Acidovorax ebreus T	0.07	0.16	5.16	42.72	0.00	0.00	0.00	0.04	2.17	0.56
taxid 573060: Acidovorax delafiel	0.08	0.16	3.10	10.16	0.00	0.00	0.01	0.06	1.21	0.76
taxid 643561: Acidovorax avenae s	0.27	0.75	3.29	10.41	0.00	0.00	0.01	0.06	4.75	0.71
taxid 391600: Brevundimonas sp B	0.13	0.60	6.57	47.20	0.00	0.00	0.00	0.03	6.42	0.62
taxid 633149: Brevundimonas subvi	0.06	0.30	8.63	82.83	0.00	0.00	0.00	0.02	3.94	0.53
taxid 1006551: Klebsiella oxytoca	0.01	0.07	18.77	451.12	0.00	0.00	0.00	0.00	1.67	0.13
taxid 1049565: Klebsiella pneumon	0.02	0.07	8.50	116.90	0.00	0.00	0.00	0.02	1.19	0.41
taxid 1125630: Klebsiella pneumon	0.00	0.01	11.19	137.82	0.00	0.00	0.00	0.00	0.07	0.05
taxid 1191061: Klebsiella oxytoca	0.03	0.09	9.17	113.05	0.00	0.00	0.00	0.02	1.36	0.33
taxid 1193292: Klebsiella pneumon	0.00	0.01	23.27	597.94	0.00	0.00	0.00	0.00	0.22	0.01
taxid 272620: Klebsiella pneumoni	0.00	0.01	9.52	106.35	0.00	0.00	0.00	0.00	0.12	0.09
taxid 296591: Polaromonas sp JS6	0.03	0.07	3.29	10.56	0.00	0.00	0.00	0.01	0.49	0.58
taxid 338969: Rhodoferax ferrired	0.01	0.03	3.23	10.88	0.00	0.00	0.00	0.00	0.18	0.40
taxid 365044: Polaromonas naphtha	0.04	0.10	3.28	10.30	0.00	0.00	0.00	0.01	0.58	0.50
taxid 395495: Leptothrix cholodni	0.40	1.15	3.28	10.29	0.00	0.00	0.01	0.05	7.24	0.71
taxid 399795: Comamonas testoster	0.07	0.19	9.15	137.85	0.00	0.00	0.00	0.08	3.64	0.63
taxid 484021: Klebsiella pneumoni	0.00	0.01	27.57	805.27	0.00	0.00	0.00	0.00	0.23	0.02
taxid 507522: Klebsiella pneumoni	0.01	0.08	16.51	317.71	0.00	0.00	0.00	0.00	1.89	0.21
taxid 525919: Anaerococcus prevot	0.06	0.79	25.03	681.33	0.00	0.00	0.00	0.00	22.39	0.25
taxid 561177: Anaerococcus hydrog	0.02	0.27	28.73	863.83	0.00	0.00	0.00	0.00	8.18	0.24
taxid 640131: Klebsiella variicol	0.00	0.01	10.83	151.91	0.00	0.00	0.00	0.00	0.17	0.11
taxid 688245: Comamonas estoster	0.04	0.10	3.48	17.02	0.00	0.00	0.00	0.03	0.88	0.57

[1] Mean relative abundance

[2] Percent frequency among all samples

Table S9: PCR primer for full length 16S rDNA amplification. Sequences of barcodes are in lowercases. Sequences of 16S rDNA parts of primers are in upper case bold.

Name	Barcode	Sequence
16S_PB01F_F27	1	tcagacgatgcgtcat**GRAGAGTTTGATYMTGGCTCAG**
16S_PB02F_R1492	2	ctatacatgactctgc**TACGGYTACCTTGTTACGACTT**
16S_PB03F_F27	3	tactagagtagcactc**GRAGAGTTTGATYMTGGCTCAG**
16S_PB04F_R1492	4	tgtgtatcagtacatg**TACGGYTACCTTGTTACGACTT**
16S_PB05F_F27	5	acacgcatgacacact**GRAGAGTTTGATYMTGGCTCAG**
16S_PB06F_R1492	6	gatctctactatatgc**TACGGYTACCTTGTTACGACTT**
16S_PB07F_F27	7	acagtctatactgctg**GRAGAGTTTGATYMTGGCTCAG**
16S_PB08F_R1492	8	atgatgtgctacatct**TACGGYTACCTTGTTACGACTT**

Table S10: Primers for Bio-Rad QX200 droplet digital 16S rDNA PCR.

Name	Sequence
F357	CCTACGGGAGGCAGCAG
R534	ATTACCGCGGCTGCTGG

Table S11: Logistic regression of Acidovorax controlling for sequence quality scores.

SCC v AD	Estimate	Std.Error	z value	Pr(>|z|)
(Intercept)	-1.3635	0.530395	-2.570724	0.010149
**Acidovorax**	4.806122	1.825424	2.63288	**0.008466**
**AvgPhred**	-3.45342	1.538986	-2.243958	**0.024835**
Phred>25	0.16372	0.349933	0.467861	0.639884
**Phred>35**	0.563349	0.12042	4.678209	**2.9E-06**
female	-0.290392	0.31125	-0.932986	0.350827
African_American	-0.025326	0.306404	-0.082655	0.934126
Stage_II	-0.055196	0.317039	-0.174098	0.861788
Stage_III	-0.266715	0.4448	-0.599627	0.548755
Stage_IV	0.584175	1.068323	0.546815	0.584506
Site_RLL	-0.45828	0.483855	-0.947143	0.343566
**Site_LEFT**	1.660212	0.676777	2.453115	**0.014163**
Site_RIGHT	0.492856	0.482733	1.020971	0.307268
Site_LUL	0.209467	0.38049	0.550518	0.581964
Site_LLL	0.563015	0.463073	1.215823	0.224053
Site_aRML	-0.498498	0.690295	-0.722151	0.470201
Smoke_Former	0.287877	0.431848	0.666617	0.505017
Smoke_Current	0.602681	0.426746	1.41227	0.157871
